# Spectroscopic and Biophysical Interaction Studies of Water-soluble Dye modified poly(o-phenylenediamine) for its Potential Application in BSA Detection and Bioimaging

**DOI:** 10.1038/s41598-019-44910-z

**Published:** 2019-06-12

**Authors:** Ufana Riaz, S. M. Ashraf, Sapana Jadoun, Vaibhav Budhiraja, Prabhat Kumar

**Affiliations:** 1 Materials Research Laboratory Department of Chemistry, Jamia Millia Islamia, New Delhi, 110025 India; 2 Advanced Instrumentation Research Facility, Jawaharlal Nehru University, New Delhi, 110067 India

**Keywords:** Materials science, Nanoscience and technology

## Abstract

Ultrasound-assisted synthesis of water soluble poly(o-phenylenediamine) (POPD) and its doping with Acid Orange (AO), Fluorescein (Fluo) and Rhodamine-6G (R6G) dyes was carried out with a view to enhance the photophysical properties of POPD. XPS studies confirmed that doping of POPD occured through hydrogen bonding between NH group of POPD and C=O/SO^−^, S=O groups of the dyes. The presence of strong hydrogen bonding was also confirmed via UV-vis studies by the addition of urea and sodium chloride to the dye modified POPD adducts. Molar extinction coefficient of these adducts was found to bear a close relationship with the molecular structure. Fluorescence life time, (τ_f,_) was found to be lowest (1.8 ns) for AO-POPD and highest (3.2 ns) for Fluo-POPD. The structure of AO-POPD was more strained, while that of Fluo-POPD was least strained. Intrinsic fluorescence decay constant, (k^0^_f_) showed increasing values for POPD, AO-POPD, Fluo-POPD, R6G-POPD as 0.071, 0.072, 0.153, and 0.172 (10^8^ s^−1^), which could be correlated to the increasing strain-free molecular structure of the adducts. Circular dichroism spectra (CD) of BSA in presence of POPD and R6G- POPD revealed that it partially broke its helical structure, while Fluo-POPD and AO-POPD showed enhancement in the helical content. The 3-D fluorescence studies confirmed enhancement in hydrophobicity of POPD and R6G- POPD and increase in hydrophylicity of AO-POP and Fluo-POPD in the microenvironment of tryptophan residue-213 of BSA. Fluo-POPD and R6G-POPD adducts were chosen to find out the lowest detection limit (LOD) of BSA by differential pulse voltammetry (DPV) which was found to be 1.35 nM, and 1.65 nM using Fluo-POPD and R6G -POPD respectively. The binding constant of BSA with Fluo-POPD- and R6G-POPD was obtained as 3.98 × 10^6^ Lmol^−1^ and 5.27 × 10^2^ Lmol^−1^. These polymers could therefore, be used for the detection of BSA. Live cell imaging revealed that POPD nanoparticles were bound to the outer membrane of *E*. *coli*, while R6G-POPD, showed penetration into the cytoplasm and excellent labeling of *E*. *coli*. This facile technique could be used to design tunable biomarkers by tailoring the conjugated polymer with a desired dye molecule.

## Introduction

Tagging of biomolecules with organic dyes, quantum dots, carbon nanotubes etc. has been one of the most promising techniques to explore the complexity and dynamics of biological interactions of multiple proteins and cells within an organism^1,2^. However, the commercialization of these nanomaterials is subjected to various limitations such as narrow excitation spectra, aggregation in water and photo-bleaching which could be circumvented by the use of conjugated polymers^3,4^. The design of fluorescent probes using conjugated polymer nanoparticles has attracted attention due to their ease of functionalization, tunable photoluminescence properties and negligible cytotoxicity^5,6^. *Chiu et al*.^5^ investigated the surface functionalization and biomolecular conjugation of semiconducting polymer dots while *Bangal et al*.^7^ developed bright, photostable, Nile Red (NR) encapsulated poly-N vinylcarbazole (PVK) fluorescent nanoparticles. It was found that the dye modified polymer showed a 15-fold enhancement in the photostability as compared to free dye molecules in water and could be safely used for bioimaging. *Liao et al*.^8^ designed near infrared emitting semiconducting copolymer dot of POPD–Rhodamine B which was utilized as an ultrasensitive fluorescence probe for the detection of NO_2_^−^ under *in-vivo conditions*. *The same authors also reported the synthesis of o*-phenylenediamine-*m*-phenylenediamine copolymer dots (omCPs) for rapid semi-quantitative detection system for Cu^2+^ ions. The copolymer dots revealed a tunable detection limit of 5–7 orders of magnitude based on the solid state fluorescence of omCPs^9^. The copolymer was also investigated as a fluorescent probe for the selective and sensitive detection of Cr^6+^ ion with a detection limit of 1 × 10^−11^ M^10,11^.

Studies reveal that POPD has been extensively investigated as a fluorescent probe for the ultrasensitive as well as selective detection for metal ions but till date no work has been reported on the modification of the photophysical properties of POPD through dye doping which could be utilized for the detection and imaging of biological analytes^12–15^. The present work therefore reports ultrasound-assisted doping of POPD with Acid Orange (AO), fluorescein (Fluo) and Rhodamine 6G (R6G) with a view to study the spectral, morphological and photophysical characteristics of POPD upon doping with dyes. Xanthenes as well as azo dyes were chosen with an intention to explore the effect of xanthene and azo groups on the fluorescence emission of POPD and the extent to which the fluorescent properties of POPD could be tuned by doping with these dyes. Bovine serum albumin (BSA) was chosen as a model protein for sensing as well as imaging studies because it is a major constituent of blood plasma exhibiting a stable 3-D structure with a number of α-helixes, β-sheets and random coils^16–21^. Interaction of POPD, R6G-POPD, AO-POPD, and Fluo-POPD with BSA was explored using fluorescence spectroscopy to obtain information about BSA’s region specific structural change, binding sites, and the nature of binding with POPD as well as dye modified POPDs. POPD, Fluo-POPD and R6G-POPD were also evaluated for their effectiveness in sensing of BSA and bioimaging of bacteria using *Escherichia Coli* (*E*. *coli)* as model system.

## Experimental

o-phenylenediamine (OPD), Rhodamine 6G (R6G), Fluorescein (Fluo), Acid Orange (AO) 1- potassium dichromate, methyl-2-pyrrolidone (NMP), and tetrahydrofuran (THF) were obtained from Sigma Aldrich, USA and were used without further purification.

### Ultrasound-assisted synthesis of poly(o-phenylenediamine)

A solution of potassium dichromate (2.72 g, 9.25 × 10^−3^ mol) was prepared in distilled water (150 ml) in a conical flask (250 ml) and o-phenylenediamine monomer (1 g, 9.25 × 10^−3^ mol) was added to the conical flask keeping the monomer:oxidant mol ratio as 1:1. The flask was sonicated for 2 h at 25 °C. The reaction mixture was then kept in deep freezer for 24 h at −20 °C. The synthesized polymer was centrifuged and washedwith deionized water to remove the unreacted monomer and oxidant and dried in vacuum oven at 70 °C for 72 h to ensure removal of water and impurities.

### Modification of the POPD with rhodamine 6G, acid orange, and fluorescein

Aqueous solutions of Rhodamine-6G (R6G), Acid orange (AO), Fluorescein (Fluo) dyes (10^−3^ M, 150 ml) were prepared and POPD (250 mg) was added to these dye solutions and sonicated for 3 h maintained at 25 °C. The modified polymers were separated via centrifugation and dried in vacuum oven for 72 h at 70 °C to ensure complete removal of water and other impurities. The % yield of POPD and dye modified POPDs were found to be ranging between 91–96%. The intrinsic viscosities (η) of POPD, R6G-POPD, AO-POPD, and Fluo-POPD were determined in NMP solution at 25 °C using Ubbehlode viscometer and were found to be 0.39, 0.47, 0.45 and 0.44 respectively. The molecular weight was determined by GPC using the protocol reported in our earlier studies^15^. The weight average molecular weights M_w_ were obtained as 10,537, 18,620, 16,323 and 15,423 for POPD, AO-POPD, Fluo-POPD and R6G-POPD respectively.

## Characterization

### Spectral analysis

FT-IR spectra of POPD and dye modified POPDs were recorded on FTIR spectrophotometer model Shimadzu IRA Affinity-I. The integrated absorption coefficient $\int \mathrm{ad}\bar{{\rm{\nu }}}$ was determined as per reported method^15^. UV-visible spectra were taken on UV-visible spectrophotometer model Shimadzu UV-1800 using water as solvent. UV-vis measurements were recorded using optical path lengths ranging between 1.0 and 10^−2^ cm for solutions containing dye concentrations ranging between 10^−3^ M–10^−5^ M to have absorbance values in the range of 0.1–1.0. The polymer concentration was taken moles per liter in all experiments. Fluorescence measurements were recorded on Fluorescence Spectrophotometer model Fluorolog®3 (Horiba Scientific, Japan) in the wavelength range of 450–700 nm (λ_exc_ = 440 nm), with excitation and emission slits of 1 nm. Solutions of dyes prepared in water in the presence/absence of polymers were measured at concentrations of 10^−5^ M while that of polymers was measured in 10^−3^ M. Life time measurements were recorded on Edinburgh FL920 Fluorescence Life Time Spectrometer and data was analyzed using FAST Software. Fluorescence decay constant, k_f_, intrinsic fluorescence decay constant, k_f_^0^, internal conversion constant, k_IC_, were all determined from decay time (τ) values^22^.

### Morphological analysis

XPS spectra were recorded on Perkin-Elmer Model 5300 XPS spectrometer equipped with a hemi-spherical electron energy analyzer, and a non-monochromatized Al(Kα) X-ray source, yielding photons of 1486.6 eV. The deconvolution of the XPS peaks and quantitative interpretation were made using the Shirley method. The curve-fitting program of Origin 2018b was used for determining Gaussian\Lorentzian ratio, the full width at half maximum (FWHM), the position and intensity of the contribution. These parameters were optimized giving the best fit to the experimental data. High resolution scans with a good signal ratio were obtained in O(1 s) and N(1 s). The quantitative analysis was based on analysis of the integral intensity (peak area) of signals measured for O(1 s) and N(1 s). High resolution transmission electron micrographs (HR-TEM) were taken on TECNAI 200 Kv TEM (Fei, Electron Optics. The HR-TEM of POPD, AO-POPD, Fluo-POPD and R6G-POPD were carried out by dispersing them in ethanol/water mixture (50:50 v/v) while the HR-TEM of BSA, POPD-BSA and dye-POPD –BSA adducts were taken in phosphate buffer saline (PBS) (pH 7.4).

### Quenching studies with bovine serum albumin (BSA)

For BSA quenching studies, POPD solution (2.0 × 10^−5^ M) was prepared in PBS (pH = 7.4, composition: NaCl (0.137 M), Na_2_HPO_4_ (0.01 M), KCl (0.0027 M), KH_2_PO_4_ (0.0018 M)). BSA (Sigma Aldrich, USA)) and BSA stock solution, (5 × 10^−6^ M) was prepared by dissolving an appropriate amount of BSA in PBS. BSA (0.35 mL, 5 × 10^−6^ M) was mixed with 0.05 ml–0.30 ml of POPD, AO-POPD, Fluo-POPD and R6G-POPD (2.0 × 10^−5^ M) respectively. All of above solutions were shaken for 30 min in an orbital shaker at 25 °C and the fluorescence emission spectra were recorded in the wavelength range of 300–550 nm on Fluorescence spectrophotometer (Fluorolog®3, Horiba Scientific) upon excitation at 380 nm. Rayleigh Light scattering (RLS) measurements of POPD, AO-POPD, Fluo-POPD, R6G-POPD prepared in PBS (2.0 × 10^−5^ M) and BSA (5.0 × 10^−6^ M) were performed by simultaneously scanning the excitation and emission monochromators of the spectrofluorometer from 50 nm to 400 nm with ∆λ = 0 nm and a slit width of 1.0 nm. Three-dimensional fluorescence spectroscopy was conducted using the above prepared concentration of POPD and dye-POPD adducts (0.3 ml, 2 × 10^−5^ M) with BSA in PBS (0.35 ml, 5 × 10^−6^ M) at scanning excitation wavelength (210 nm–310 nm) and emission wave length (200 nm–800 nm) with an increment of 10 nm, and slit width of 2.5 nm on F-2500 fluorescence spectrophotometer (Hitachi, Japan). For circular dichroism (CD) measurements, BSA in PBS (0.5 ml, 5.0 × 10^−6^ M) taken as control was mixed with 2.5 mL of POPD (2.0 × 10^−5^ M) and similar test solutions of BSA with AO-POPD, Fluo-POPD, and R6G-POPD were prepared. All of the above solutions were shaken for 30 min immediately after mixing at room temperature. The measurements were taken in the UV region in the wavelength range of 195 nm–230 nm with band width of 5 nm on a Jasco- 815 automatic recording spectro- polarimeter (Japan) in a cell of 2 mm path length at room temperature. Dry nitrogen gas was used to purge the machine before and during the measurements. Fluorescence images of the BSA adduct of POPD and the above modified polymers were obtained using a Laser Confocal Microscope with Fluorescence Correlation Spectroscopy (FCS)-Olympus FluoView FV1000 equipped with a He−Ne laser and oil immersion objective. λ_max_ for laser excitation was 410 nm.

### Electrodeposition and electrochemical detection of BSA using POPD and dye doped POPDs

For the electrophoretic deposition of POPD, Fluo-POPD, R6G-POPD-R6G onto ITO electrode, samples were diluted in ethanol (1:4) keeping the concentration 0.1 mg/mL. This solution was connected with potentiostat using a two-electrode system (a pre-hydrolyzed ITO electrode acting as cathode with parallel-placed platinum acting as the anode) and DC potential of 11 V was applied for 30 sec to obtain the electrodes. The electrochemical studies of the fabricated electrodes were performed using Autolab potentiostat/galvanostat (Eco Chemie, Netherlands) using a three-electrode cell. POPD and dyes doped POPD based Indium Titanium Oxide (ITO) were used as working electrode, platinum wire as auxiliary electrode and Ag/AgCl as reference electrode. BSA dissolved in PBS (100 mM, pH 7.4, 0.9% NaCl) containing 5 mM [Fe(CN)_6_]^3−/4−^ was utilized in conducting the experiments. The effect of pH on the electrodes were investigated ranging from 6.0 to 8.0 at 25 °C. The results observed at pH 7.4 shows the higher current response. Therefore, all the experiments were performed at pH 7.4.

### Live cell imaging studies

*E*. *coli* (MTCC 443) was procured from MTCC (Chandigarh, India) for bioimaging studies and was grown in LB media at 37 °C until it reached an approximate value of 10^9^ cfu/mL (plate-counting method). The bacteria solution was collected and centrifuged for 30 min at 4 °C. Sterile phosphate-buffered saline (PBS, pH 7.2) solution was used to wash *E*. *coli* cells three times. Aqueous solutions of the POPD, AO-POPD, Fluo-POPD, and R6G-POPD (50 μL,5 mg/ml) were added to PBS solution (5 ml) of *E*. *coli* cells with OD 1.0. The suspensions were gently shaken and incubated for 30 min at 25 °C with occasional shaking and were centrifuged at 10000 rpm for 10 min. The adducts obtained were washed three times with PBS solution and redispersed in 5 ml PBS solution. The polymer bound bacteria were observed using Nikon Real Time Laser Scanning Confocal Microscope Model A1R with motorized inverted microscope having Live Cell and Spectral Imaging (Model Ti-E). The POPD and dye doped POPD –labeled bacteria were detected using an excitation wavelength of 488 nm. Emitted light was collected through 480 nm dichroic filter, a 520 nm long pass filter, and a 680 nm short-pass filter. Three-dimensional reconstruction of images from multiple optical sections was carried out using NIS Software. Optical sections for surfaces were collected by setting the stepping motor increment and the pixel size at ca. 0.3 mm.

## Results and Discussion

### Confirmation of doping of POPD with dyes using XPS and IR analysis

**Figure 1:**
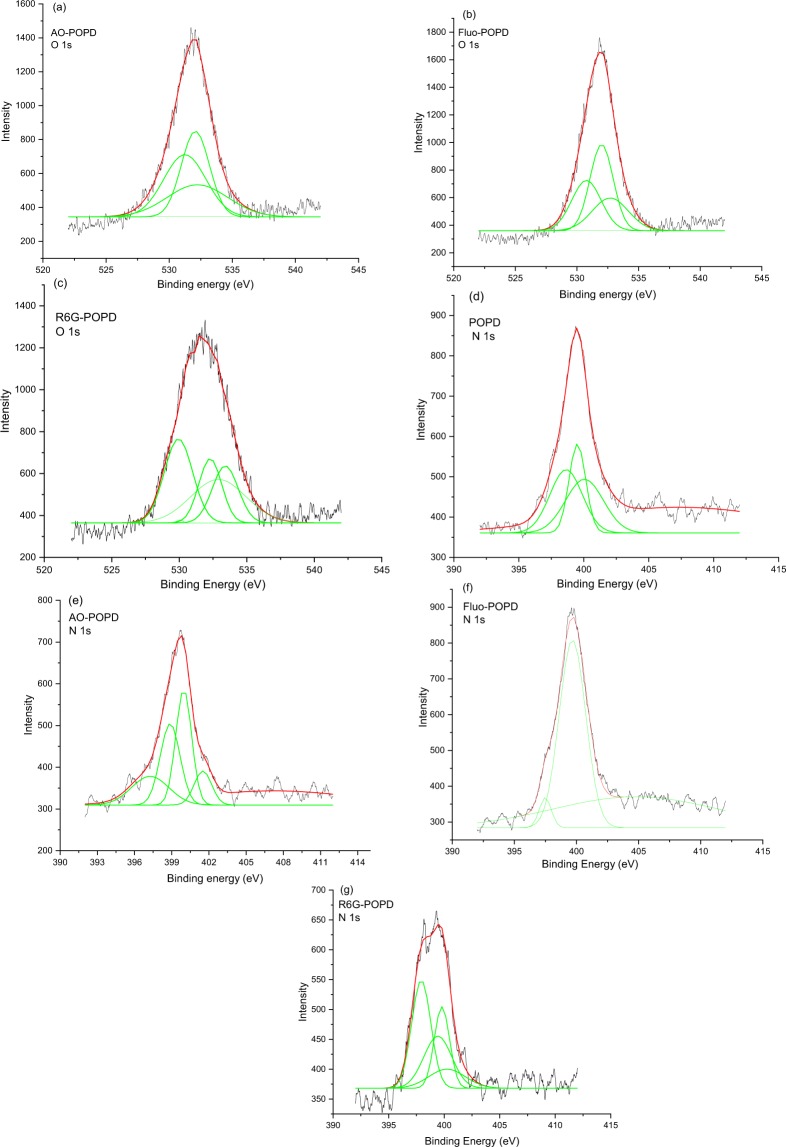
XPS spectra of deconvoluted region of (**a**) AO-POPD (O 1 s), (**b**) Fluo-POPD (O 1 s), (**c**) R6G-POPD (O 1 s), (**d**) POPD (N 1 s), (**e**) AO- POPD (N 1 s) (**f**) Fluo-POPD (N 1 s), (**g**) R6G–POPD (N 1 s).

**Figure 2:**
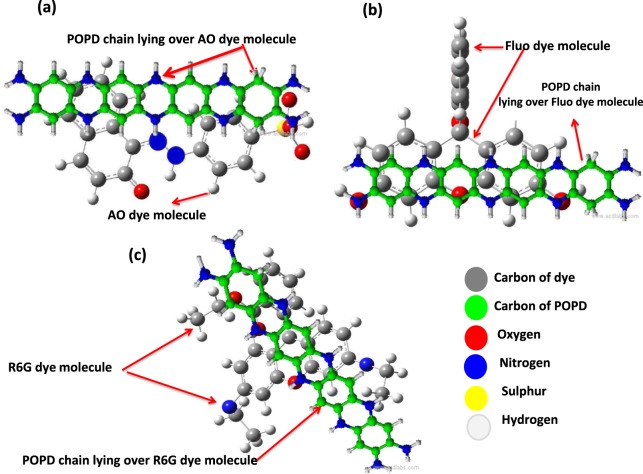
Chemical structures projecting the orientation of POPD in (**a**) AO-POPD, (**b**) Fluo-POPD and (**c**) R6G-POPD.

The doping of POPD by AO, Fluo and R6G dyes was confirmed by via XPS spectroscopy. The binding energy (B.E) spectra of N 1 s and O 1 s of POPD and dye-POPD adducts were recorded and are shown in Fig. 1(a–g). The doping of POPD by AO dye occured through the interaction of SO_3_^−^ of AO group with the NH group of POPD, Fig. 2(a). As both SO_3_^−^ group in AO and POPD are planar, the NH group will lie over the SO_3_^−^ group of AO dye. Fluorescein interacted through the C=O of xanthene ring which is in the same plane as the –NH– group of POPD, Fig. 2(b). POPD will lie over the xanthene ring and can undergo π-π interaction between the xanthene ring of Fluo and benzene ring of POPD. R6G interacted through C=O group of the ester attached to aromatic ring, and also through the ether (–O–). group, Fig. 2(c). The planar POPD chains lie over the R6G molecule in a lateral mode. It is expected that electron density over O will slightly decrease consequent to H-bonding. The B.E. values of O 1 s peak of these moieties will therefore increase upon interaction as electron density around O will decrease. We observed that O 1 s B.E. peak of SO^−^ (530 eV) and S=O (531.19 eV and 531.68 eV) increased to 532.4 eV and 532.6 eV, Figs 1(a) and S1 (provided in supplementary information). This clearly established definite binding of AO with POPD. Fluorescein showed B.E. peak associated with O 1 s of –C=O of planar xanthene ring at 532.11 eV, Fig. S1(b). Its interaction with POPD pushed the binding energy peak to 532.4 eV, Fig. 1(b). These changes indicated strong binding of Fluo with POPD. Likewise B.E. of O 1 s in C=O of ester group of R6G was pushed from 533.6 eV to 535 eV upon interaction with POPD, Fig. 1(c)^23^. It can be argued that nitrogen in –NH– group is less electron rich than in the –NH_2_ group, hence the B.E. of N 1 s of the former will be higher than later. Pure POPD showed N 1 s B.E. peak of –NH– at 400 eV, Fig. 1(d). Since hydrogen bonding causes depletion of electrons in N, its B.E. will slightly increase than parent N. Thus the B.E peak at 400 eV associated with –NH– was pushed to 401.04 eV in AO-POPD, Fig. 1(e) which indicated strong binding of POPD with AO. In Fluo-POPD, the B.E peak of N 1 s of POPD, 400.06 eV Fig. 1(d) was pushed up to 404.7 eV, Fig. 1(f). The foregoing values showed strong interaction of POPD and Fluo. We have already shown that C=O of R6G will interact with –NH– of POPD. The N 1 s B.E. peak of –NH– of POPD, Fig. 1(g) was found to move from 400.06 eV to 400.25 eV. This indicated binding with POPD but a little weak one apparently because of lateral overlying of POPD molecule on R6G shown in Fig. 2(c). The above results confirmed doping of POPD by dyes.

IR spectrum of POPD, (Given as Supplementary Information, Fig. S2), revealed NH stretching band at 3390 cm^−1^ and a shoulder at 3157 cm^−1^. The NH-imine stretching for POPD appeared at 1646 cm^−1^, while CN stretching vibration was observed at 1369 cm^−1^. The peaks corresponding to ring puckering of quinonoid and benzenoid appeared at 1531 cm^−1^ and 1483 cm^−1^ respectively. The presence of the above peaks confirmed the polymerization of POPD^15^. Upon modification of POPD with AO, the IR spectrum of the polymer revealed a shift in the NH stretching vibration peak from 3390 cm^−1^ to 3473 cm^−1^, while the NH peak area appeared to be highly reduced confirming the interaction of AO with POPD. The quinonoid and benzenoid peaks also shifted from 1531 cm^−1^ and 1483 cm^−1^ respectively in case of pristine POPD to 1570 cm^−1^ and 1489 cm^−1^. The presence of S–O stretching and C–OH stretching peaks at 1240 cm^−1^ and 1038 cm^−1^ confirmed the presence of AO in POPD. Likewise the spectrum of Fluo-POPD showed an increase in the NH stretching vibration peak to 3446 cm^−1^ while the imine stretching peak appeared to be highly pronounced. The quinonoid and benzenoid appeared to be suppressed. The spectra of R6G-POPD showed the NH stretching peak at 3409 cm^−1^. The benzenoid and quinonoid peaks also revealed a shift of 40 cm^−1^ and 62 cm^−1^ respectively_._ It was observed that the intensities of the NH stretching vibration peak as well as the benzenoid quinonoid peaks revealed either enhancement or reduction upon dye modification. The absence of peaks associated with the dyes indicated that the product was not a mixture of polymer-dye and therefore further confirmed the doping of POPD by dyes.

### Optical characteristics of POPD and dye-POPD adducts

**Figure 3:**
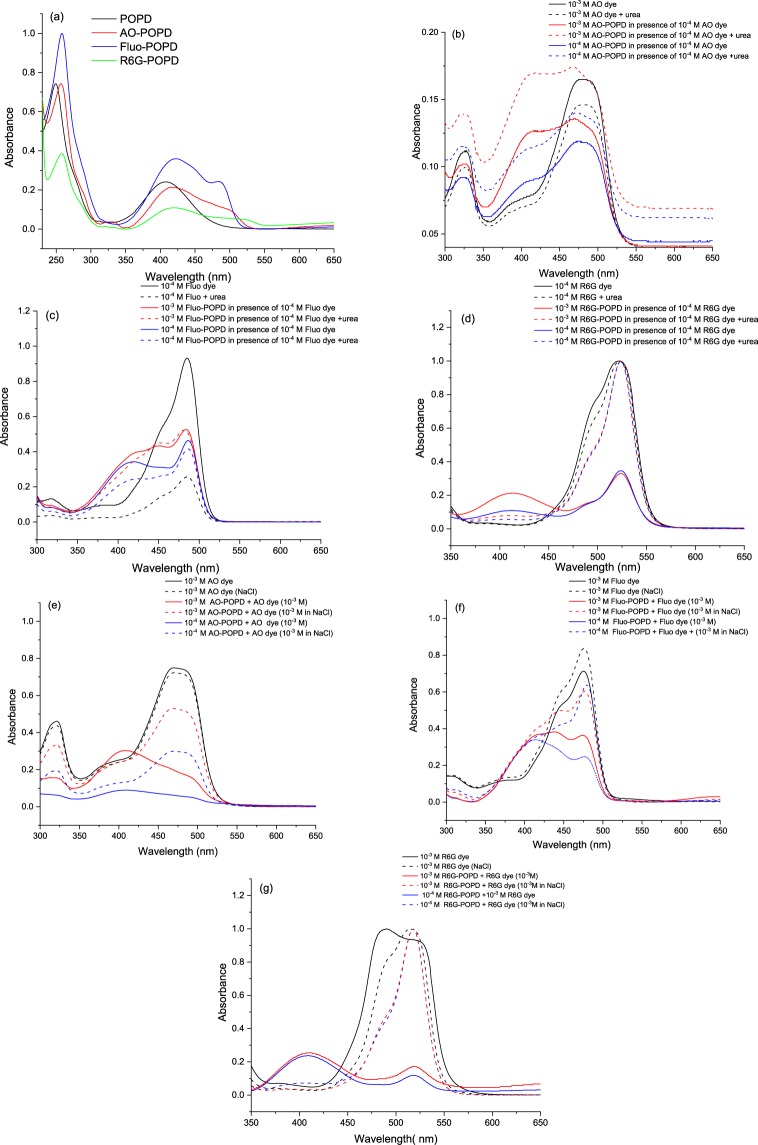
(**a**) UV-visible spectra of (**a**) POPD and dye-POPD adducts in water (10^−3^ M) (**b**) effect of addition of urea to AO-POPD, (**c**) effect of addition of urea to Fluo-POPD (**d**) effect of addition of urea to R6G-POPD (**e**) effect of addition of NaCl to AO-POPD (**f**), effect of addition of NaCl to Fluo-POPD (**g**) effect of addition of NaCl to R6G-POPD.

The UV –visible spectra were recorded for pristine dyes, POPD, and dye-POPD adducts to explore the phenomena of binding, interaction and structural changes, Fig. 3(a), (Supplementary Information Fig. S3(a–c)). Since xanthene and azo dyes were used as dopants for the polymer, the presence of hydrogen bonding and π-π interactions in these systems were further confirmed by the use of electrolytes such as NaCl and urea, Fig. 3(b,c). It is well known that xanthene dyes such as R6G and Fluo undergo aggregation at increasing concentrations to form dimers and higher order aggregates and this molecular aggregation significantly transforms the absorption characteristics of the dye. Therefore, the UV visible spectra of AO, Fluo and R6G dyes were taken from 10^−3^ M–10^−5^ M to explore the concentration at which these dyes undergo self-aggregation (given in Supplementary Information Fig. S3(a–c)). The UV-visible spectrum of AO dye (given in Supplementary Information, Fig. S3(a)) revealed two peaks; one at 385 nm associated with the presence of naphthalene ring and the other at 475 nm that was correlated to the presence of monomer azo (N=N) bond. With the increase in the dye concentration the peaks revealed only an increase in intensity. The UV spectrum of Fluo dye (given in Supplementary Information, Fig. S3(b)) revealed an intense peak at 460 nm and a shoulder at 435 nm. The peak at 460 nm was associated with the presence of monomer band while the shoulder centered at 425 nm was correlated to the dimer band. With increasing dye concentration, the intensity of the dimer band increased due to the formation of H-type contacts due to self-aggregation as confirmed by other authors^16,24^. Similarly, the UV-visible spectrum of R6G dye (given in Supplementary Information, Fig. S3(c)) revealed monomer band centered at 520 nm and the dimer band at 480 nm. The intensity of the dimer band decreased with the decrease in the dye concentration and was found to be negligible at the concentration of 10^−5^ M. The UV spectra of POPD and dye-POPD adducts (taken in concentrations of 10^−4^ M) are depicted in Fig. 3(a). Pristine POPD revealed two major peaks, a small peak at 250 nm and a well-formed peak at 415 nm. The former peak was correlated to the π-π* transition while the later peak was associated with polaronic transitions in POPD^15^. Upon doping of POPD with AO, a broad hump appeared spanning between 400 nm–500 nm which was due to the merging of large peak of POPD centered at 415 nm with a suppressed peak of AO dye at 475 nm. Similarly upon doping of POPD with Fluo, Fig. 3(a), the well-formed polaronic transition peak of POPD was observed at 425 nm while a small peak was noticed at 490 nm which was correlated to the monomeric peak of Fluo dye. Likewise, the UV spectrum of R6G-POPD exhibited a pronounced hump around 420 nm of POPD and a suppressed broad peak at 520 nm of R6G. In all the dye doped adducts, a red shift of 25–30 nm in dye peaks was observed. Doping therefore modified the electronic structure (LUMO) of dyes. It was also observed that peaks of dyes in all the dye-POPD adducts were much suppressed than the POPD peak indicating that dyes were much smaller in amount in doped POPD, Fig. 3(a). The above mentioned facts confirmed the doping of POPD by respective dyes. From the calibration curves of pristine dyes and doped POPD adducts, the loading of dye in POPD was calculated to be 0.50 mg for AO-POPD, 1.04 mg for Fluo-POPD and 1.05 mg for R6G-POPD (per mg of pristine POPD), percent doping being 5%, 10.4%, and 10.5%.

It was revealing to explore the effect of adding POPD in dyes solutions. The addition of increasing amounts of POPD (10^−4^ M) to AO dye solution (10^−5^ M) (given in Supplementary Information as Fig. S4(a)), revealed that the intensity of AO monomer peak decreased substantially from 1.25 to 0.3. Likewise intensity of dimer peak also decreased. The addition of AO-POPD to AO dye solution also showed reduction in the intensity of the AO monomer and dimer peaks of AO dye, (given in Supplementary Information as Fig. S4(d)). Interestingly, the POPD transition peak around 410 nm did not appear at all in both the systems. It appears that AO dye strongly binds to POPD and as a result both AO and POPD are structurally deformed. The amount of active dye goes on decreasing and the absorption intensity reduces to lower values which indicate a strong binding of AO dye with POPD. Similar behavior was also observed with Fluo dye (given in Supplementary Information as Fig. S4(b)). In this case also, addition of POPD to Fluo dye decreased the monomer absorption peak of Fluo substantially from 0.9 to 0.4. Similarly, the addition of Fluo-POPD to Fluo dye solution revealed reduction in the monomer peak intensity from 0.98 to 0.6 (given in Supplementary Information as Fig. S4(e)). The structure of POPD appears to be strained and undergoes deformation at the same time and therefore its transition peak does not appear. A strong binding between Fluo and POPD is therefore confirmed. Interestingly, the addition of POPD to R6G dye, reduced the monomer peak considerably from 0.85 to 0.5, while the POPD transition peak about 410 nm appeared even with addition of small amount of POPD solution,(given in Supplementary Information as Fig. S4(c)). Similar trend was observed upon adding R6G-POPD to R6G dye solution, (given in Supplementary Information as Fig. S4(f)). This behavior was found to be strikingly different from the previous two dyes. It showed that the mode of binding of R6G with POPD was different from that of AO and Fluo dyes.

The effect of addition of dye solutions to POPD and dye doped POPDs was also investigated (given in Supplementary Information as Fig. S5(a–f)). The addition of AO dye solution to POPD and AO-POPD significantly suppressed the peak corresponding to POPD while the monomer peak of the AO dye revealed an increase in its intensity, (given in Supplementary Information as Fig. S5(a,d)). Likewise the addition of Fluo dye solution to POPD and Fluo-POPD suppressed the POPD peak in both cases, (given in Supplementary Information as Fig. S5(b,e)) while in case of addition of R6G dye to POPD as well as R6G-POPD, a reduction in the POPD peak intensity was noticed upon increasing addition of R6G-dye solution, (given in Supplementary Information as Fig. S5(c,f)).

Optimized Gaussian structures of the three dyes, is given in Supplementary Information as Fig. S6(a–c) which shows them to be planar. The functional groups in AO and Fluo dyes S6 (a,b) are located from right to left or left to right in the molecular plane. For hydrogen bonding, planar POPD molecule will lie over AO and Fluo molecules in a parallel manner, Fig. 2(a,b). With the above configuration of POPD and AO/Fluo, some π-π interaction between the two also occurs. In case of R6G dye, the functional groups –C=O, –O– are located laterally to the molecular plane, (Fig. S6(c)). Hence POPD molecule will lie laterally but in a parallel manner over R6G, Fig. 2(c). Since POPD is a large polymeric molecule, only a small part of it will be involved in the binding and a major part of it will be free from binding, hence it shows its transition peak at about 410 nm along with R6G monomer peak. Moreover π–π interaction in this case will be weak because of the lateral juxtaposition which also helps in the appearance of POPD transition peak.

To confirm the hydrogen bonding between dye-POPD adducts, effect of addition of urea and NaCl to AO-POPD, Fluo-POPD and R6G-POPD adducts solutions was explored, Fig. 3(b–g). Urea is well known for breaking of hydrogen bonds and NaCl is known to promote aggregation of dyes and proteins^16,17^. We observed that when urea was added to AO-POPD adduct solution, the intensity of the monomer absorption peak at 475 nm was considerably enhanced than the parent adduct. Likewise, the polaronic transition peak and π-π* transition peak intensities of POPD were enhanced, Fig. 3(b). Such a remarkable change is brought about by the breaking of hydrogen bonds between AO-POPD. Consequent to breaking of hydrogen bonds, both AO and POPD are liberated and show enhanced absorption intensity. Similarly, when urea was added to Fluo-POPD adduct solution, the monomer absorption peak of Fluo at 490 nm was substantially enhanced which indicated breaking of hydrogen bond between Fluo and POPD and presence of unbound Fluo molecules, Fig. 3(c). Transition peak of free POPD was enhanced but appeared as a suppressed shoulder at about 420 nm. In case of R6G-POPD adduct, the addition of urea led to the breaking of hydrogen bond between the two and liberation of R6G molecules. The monomer peak intensity at 535 nm was highly enhanced from 0.2 a.u. to 1.65 a.u, Fig. 3(d). The transition peak intensity of POPD at 415 nm was negligibly enhanced. We have already argued that in case of POPD-R6G adduct, major part of POPD molecules do not undergo hydrogen bond formation or π-π interaction. Hence, liberation of R6G from POPD does not bring about any significant change in its structure. Therefore, the transition intensity of POPD is insignificantly changed upon addition of urea. Thus, addition of urea clearly establishes hydrogen bond formation between POPD and R6G, Fluo, and AO. Addition of NaCl to AO-POPD, Fluo-POPD, R6G-POPD solution provided enhanced electrolyte environment, Fig. 3(e–g). It was observed that addition of NaCl to pure POPD, (given in Supplementary Information Fig. S7), decreased its transition intensity to some extent because of the decrease in dielectric constant of water. Addition of NaCl to AO-POPD, Fluo-POPD, R6G-POPD adducts solution, Fig. 3(e–g), decreased the absorption intensities of AO, Fluo and R6G dyes and also the transition intensity of POPD. The hydrogen bonds between POPD and AO, Fluo, and R6G were uninterrupted. The decrease in the absorption and transition intensities was caused by the lowering of the dielectric constant of the water. *Hamlin et al*.^17^ have also found that in presence of NaCl, some anionic dyes showed noticeable decrease in their absorption intensities.

**Figure 4:**
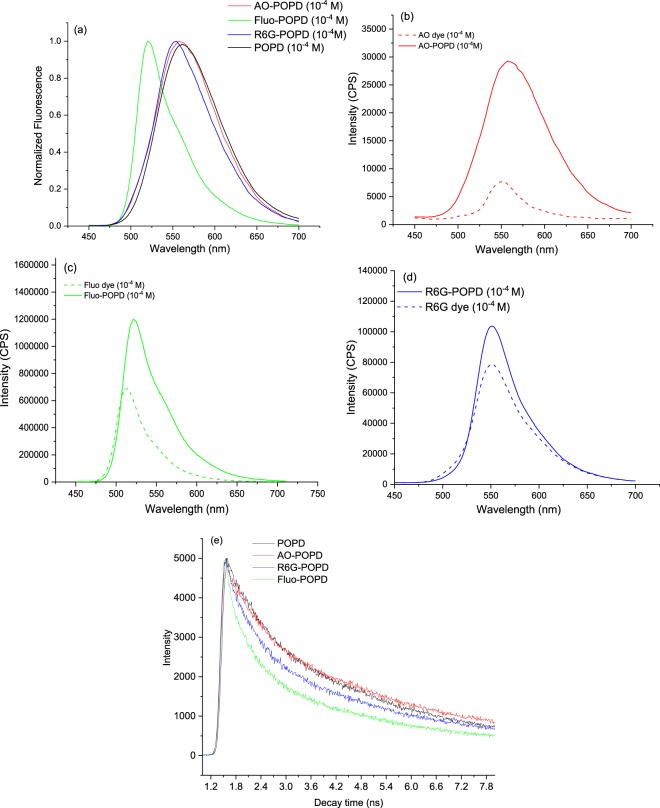
Fluorescence emission spectra of (**a**) POPD and dye-POPD adducts (**b**) AO dye and AO-POPD, (**c**) Fluo dye and Fluo-POPD, (**d**) R6G dye and R6G-POPD (**e**) time resolved spectra of POPD and dye-POPD adducts (λ_exc_= 380 nm).

The fluorescence emission spectrum of POPD and dye-POPD adducts are shown in Fig. 4(a), Upon excitation at 380 nm, POPD revealed emission peak at 560 nm which was attributed to S_1_ → S_0_ transition^15^. The λ_emis_ values AO-POPD, Fluo-POPD and R6G-POPD were observed at 558 nm, 510 nm, and 555 nm. The λ_emis_ values of AO-POPD and R6G-POPD remained quite closer to the λ_emis_ of the respective original dyes, Fig. 4(b,d) but λ_emis_ of Fluo-POPD showed shift 6 nm as compared to Fluo dye, Fig. 4(c). The corresponding λ_max_ values were found to be 410 nm, 425 nm, 480 nm and 520 nm, Table 1. This gives stokes shift of 150 nm for POPD, 133 nm for AO-POPD, but a low value of 30 nm for Fluo-POPD and 35 nm for R6G-POPD. It can therefore be concluded that excited state molecular orbital of xanthene ring undergoes only a little change vis a vis the LUMO molecular orbital, while non-xanthene moieties, POPD and POPD-AO shows a large perturbation in their excited state molecular orbitals. Comparison of the molar extinction coefficient of dye-POPD adducts are expected to reveal some aspects of their structural characteristics. POPD showed appreciable value of molar extinction coefficient ε_M_ value of 26,500 which indicated appreciable transition from HOMO to LUMO and less strained structure. AO-POPD adduct revealed ε_M_ value of 14,680 indicating hindered transition from HUMO to LUMO and strained molecular structure. POPD-R6G showed ε_M_ value of 19,400 showing less hindered transition from HOMO-LUMO and strained molecular structure. Fluo-POPD showed ε_M_ value of 27,600 higher than that of pure POPD indicating appreciable transition from HOMO-LUMO and least strained molecular structure, Table 1.

**Table 1:** Molar extinction coefficient, oscillator strength and fluorescence decay characteristics of POPD and dye-POPD adducts.

Sample concentration (10^−4^ M)	λ_max_ (nm) (UV visible)	${\boldsymbol{\int }}\mathrm{ad}\bar{{\boldsymbol{\nu }}}$ (integrated absorption coefficient) cm^−2^	Molar Extinction Coefficient (ε_M_)	Oscillator Strength	λ_emis_ (nm) (fluore scence)	I_samp_ (Integrated area) (fluore scence)	τ_f_ (ns) Obtained by Global analysis	*k_f_ (10^8^ s^−1^)	*k_f_^0^ (10^8^ s^−1^)	*k_IC_ (10^8^ s^−1^)	Quantum yield (water) (Ф)
AO	475	702	1720	0.03	553	1.46 × 10^7^	—	—	—	—	—
Fluo	467	1516	4857	0.06	516	9.17 × 10^8^	—	—	—	—	—
R6G	520	880	5150	0.04	552	1.92 × 10^8^	—	—	—	—	—
POPD	410	1349	26500	0.58	560	1.42 × 10^8^	3.1	3.22	0.071	3.14	0.23
AO-POPD	425	1245	14680	0.54	558	1.46 × 10^8^	2.2	4.54	0.072	4.46	0.16
Fluo-POPD	480	1121	27600	0.48	510	1.37 × 10^8^	1.8	5.55	0.172	5.37	0.49
R6G-POPD	520	489	19400	0.21	555	1.32 × 10^8^	3.2	3.75	0.153	3.59	0.31

Therefore, in the dye-POPD adducts, the molar extinction coefficient bears a close relationship with their unstrained structure. POPD undergoes hydrogen bond formation with SO^−^ and –N=N– in POPD-AO adduct leading to a strained structure. As shown earlier, POPD chain lies over R6G laterally and undergoes hydrogen bond formation with –C=O; this structure is less strained than the one in the previous case. The XPS studies have shown that Fluo dye forms hydrogen bond with POPD, the later overlying the former but slightly displaced off. The π-π interaction between the xanthene ring and benzene ring of the former stabilizes the structure. Fluo-POPD adduct thus has least strained structure and highest molar extinction coefficient value among the three dye-POPD adducts. Molecular structure therefore plays an important role in determining the molar extinction coefficient of these products. The photophysical constants of POPD and POPOD-dyes adducts were determined and are tabulated in Table 1. Fluorescence life time, (τ_f_,) was obtained by global analysis of time resolved data. The τ_f_ values of R6G-POPD, AO-POPD, Fluo-POPD, and POPD was respectively found as 1.8 ns, 2.2 ns, 3.2 ns and 3.1 ns, Table 1, Fig. (e). These values are related to the decay characteristics of their excited state and its structure. Low value of τ_**f**,_ of 1.8 ns cannot be explained on this basis. Variation of quantum yield values does not follow any regular pattern. Intrinsic fluorescence decay constant (k^0^_f_) takes into accounts both τ_**f**,_ and Φ which accurately indicated the decay characteristics. When the molecular structure is least strained, the **τ**_**f**_ is high. The k_f_^0^ values of POPD, AO-POPD, Fluo-POPD, and R6G-POPD in the increasing order were observed to be 0.071, 0.072, 0.153, and 0.172 (10 ^8^ s^−1^). Thus 7.1 × 10^6^ molecules of POPD, 7.2 × 10^6^ molecules of AO-POPD, 15.3 × 10^6^ molecules of Fluo-POPD and 17.2 × 10^6^ molecules of R6G-POPD are decaying per second. Increasing values of k^0^_f_ showed comparatively increasing strain free molecular structure, particularly the molecular orbital structure of the excited state. Fluo-POPD and R6G-POPD showed highest k^0^_f_ values because they have least strained molecular structure. The increasing order of the intrinsic decay constant matches with the optical characteristics and molar extinction coefficient data. Increasing values of k_f_^0^ indicated comparatively increasing unstrained molecular structure, especially molecular orbital structure of the excited state. Fluo-POPD with life time value of 3.2 ns, quantum yield value 0.49 and intrinsic decay constant value 0.153 (10^8^ s^−1^) was seen as a good candidate for use in further photophysical studies but has some solubility constraint in water. R6G-POPD with life time value of 1.8 n s, quantum yield of 3.2, decay constant value of 0.172 (10^8^ s^−1^), and λ_max_ value of 520 nm and fair solubility in water was used in photophysical investigations of relevant biological problems. We have therefore used Fluo-POPD and R6G-POPD for finding out the lowest limit of detection of BSA (a widely used protein in biological studies) through differential pulse voltammetry (DPV) which is discussed in the later section.

### Interaction of POPD and dye- POPD adducts with BSA analyzed via UV and fluorescence studies

The interaction of BSA with POPD, R6G-POPD, AO-POPD, Fluo-POPD was studied using UV spectroscopy which gives a definite indication of its binding characteristics. The absorption peak of π → π* in BSA is associated with the C=O group of polypeptide backbone which appeared at 280 nm. In presence of POPD, R6G-POPD, AO-POPD, Fluo-POPD, the absorption intensity increased considerably and a blue shift of about 12–15 nm was noticed (Given in Supplementary Information, Fig. S8(a–d)). The increase in the intensity of peak associated with BSA was attributed to conformational changes in the coiled peptide backbone of BSA upon complex formation with POPD and dye modified POPDs and also confirmed that the micro-environment around the peptide backbone was more hydrophobic^18–20^.

Fluorescence has been widely used for exploring the various aspects of biophysical interactions studies with BSA. BSA exhibits fluorescence through its tryptophan and tyrosine residues that undergo quenching upon interaction with a host comprising of endogenous and exogenous moieties. Fluorescence quenching of BSA implies structural changes, transition in micro-environment, binding with drug as well as other biologically active molecules, and denaturation. As POPD and dye modified POPDs were found to be completely water soluble, fluorescence quenching of BSA was also investigated in presence of these polymers. (given in Supplementary Information, Fig. S9(a–d)). BSA was observed to undergo quenching in presence of POPD and dye modified POPDs which occurred through molecular interactions such as ground state complex formation, electron transfer or bimolecular collision. When quenching takes place via ground state complex formation, it is called static quenching and when it occurs through collision between the quencher and excited state fluorophore, it is called dynamic quenching. Static and dynamic quenching can be differentiated by observing the effect of temperature on quenching. Dynamic quenching shows enhancement in the quenching constant values while static quenching shows a decrease in the quenching constant values with the increase in temperature^21^. The fluorescence quenching is described by the Stern–Volmer constant which is related to the bimolecular quenching rate constant, k_q_, K_SV_ = k_q_τ_0_, and τ_0_ is the average lifetime of BSA evaluated as 10^−8^ s^25–27^. Values of Stern-Volmer constant (K_SV_) at 30 °C were found to be 7.78 × 10^−5^, 7.8 × 10^−5^, 6.98 × 10^−5^, and 10.6 × 10^−5^ LM^−1^ respectively for POPD, R6G-POPD, Fluo-POPD, AO-POPD (Given in Supplementary Information, Fig. S10(a–d)). Among the dye modified polymers, quenching efficiency was found to be the highest for AO-POPD, while it was observed to be lowest for Fluo-POPD. This effect can be attributed to the intense electrostatic binding of BSA with the sulphonate and carboxyl groups present in AO-POPD. In Fluo-POPD, steric repulsion is experienced from the oxygen and C=O groups of Fluorescein dye which reduces the quenching efficiency. Quenching rate constant (K_q_) for POPD, R6G-POPD, Fluo-POPD, AO-POPD were calculated to be 7.78 × 10^−13^, 7.8 × 10^−13^, 6.98 × 10^−13^, and 10.6 × 10^−13^ LM^−1^s^−1^, respectively. The K_q_ value was found to be highest for AO-POPD and lowest for Fluo-POPD (Supplementary Information, Fig. S10(a–d)). The obtained quenching rate constant value was fairly larger than the value obtained for biological macromolecules due to the collision mechanism (2.0 × 10^10^ LM^−1^s^−1^)^27^. This showed that quenching of BSA by POPD and dye modified POPDs took place through static mechanism via the formation of an intermolecular complex. It was noted that the K_q_ value was of the order of 10^10^ LM^−1^s^−1^ in our case while in other cases, it was found to be of the order of 10^13^ LM^−1^s^−1^ and above^27^. The Stern Volmer constant (K_SV_) for POPD and dye modified POPDs was found to increase as the temperature was raised from 10 °C to 30 °C (Supplementary Information, Fig. S11(a–d)) which established the mechanism of dynamic quenching in these systems^28^. The figures revealed both static as well as dynamic quenching. It can be argued that initially quenching takes place through ground state complex formation, but upon excitation, the mechanism of dynamic quenching dominates and increases with increase in temperature. *Mariam et al*.^29^ have also reported static and dynamic quenching in a similar polymer system. The binding constant of POPD and dye modified POPDs with BSA and the number of binding sites were calculated as per equation reported in our previous studies^15^. The binding constant of these polymers were determined at 10 °C, 20 °C, 30 °C and are shown in Table 2. The binding constant values were noticed to be fairly high (of the order of 10^6^). The polymers POPD, R6G-POPD, Fluo-POPD showed almost similar values of binding constant but AO-POPD exhibited slightly higher value of binding constant due to the presence of both sulphonate and carboxyl groups in AO that facilitated hydrophobic binding as well as electrostatic binding. The ΔH and ΔS values of the interactions of POPD, R6G-POPD, Fluo-POPD, AO-POPD with BSA were determined using van’t Hoff equation^28^. The free energy (ΔG) values were found to be negative for all the polymers indicating that the interaction of POPD and dye modified POPDs with BSA was spontaneous. The ΔG values for POPD, R6G-POPD, Fluo-POPD, AO-POPD varied in close range of 40.5 KJ/mol^−1^–47.6 KJ mol^−1^. The binding sites of the polymers at 30 °C were found in the same order as 1.6, 1.8, 2.0, and 3.1 respectively, which were in agreement with the respective binding constant of these polymers. The binding sites involved are Tryptophan-213, Tyrosine, and Tryptophan-134. *Ross and Subramaniun*^20^ have shown that the nature of binding of exogenous moieties with BSA can be known if ∆H and ∆S values of interaction of BSA with such moieties are calculated. The nature of binding is as follows:

(i). ΔH > 0, and ΔS > 0, indicates hydrophobic force;
(ii). ΔH < 0, and ΔS < 0 shows van der Waals’ force and hydrogen bonding are operative;
(iii). ΔH < 0 and ΔS > 0, indicates dominant electrostatic interactions.

**Table 2:** Thermodynamic parameters and quenching constants of POPD and dye-POPD adducts.

Moieties	Temperature (°C)	K_SV_ × 10^4^	K_q_ × 10^12^	K_a_ × 10^6^	∆H^0^, KJmol^−1^	∆S°, Jmol^−1^ K^−1^	∆G^0^, KJmol^−1^	n Binding Sites
POPD-BSA	10	5.76	5.76	5.00	25.7	218.5	−40.55	1.34
	20	7.70	7.70	8.00				1.52
	30	7.78	7.78	12.50				1.60
AO-POPD-BSA	10	8.50	8.50	25.10	67.39	379.5	−47.6	2.10
	20	9.00	9.00	79.10				2.80
	30	10.60	10.60	120				3.10
Fluo-POPD-BSA	10	5.70	5.70	7.90	27.37	230.41	−42.32	1.49
	20	5.90	5.90	15.80				1.80
	30	6.98	6.98	21.80				2.00
R6G-POPD-BSA	10	5.36	5.36	8.40	22.24	210.2	−41.45	1.60
	20	7.11	7.11	8.90				1.63
	30	7.80	7.80	15.80				1.80

Table 2 reveals that both ΔH and ΔS are positive for POPD, R6G-POPD, Fluo-POPD, AO-POPD indicating that the hydrophobic interaction dominates and binding is predominantly driven by entropy.

### Conformation of structural changes in BSA upon interaction with POPD and dye-POPD adducts via resonance light scattering (RLS), circular dichroism (CD) and 3-D fluorescence studies

**Figure 5:**
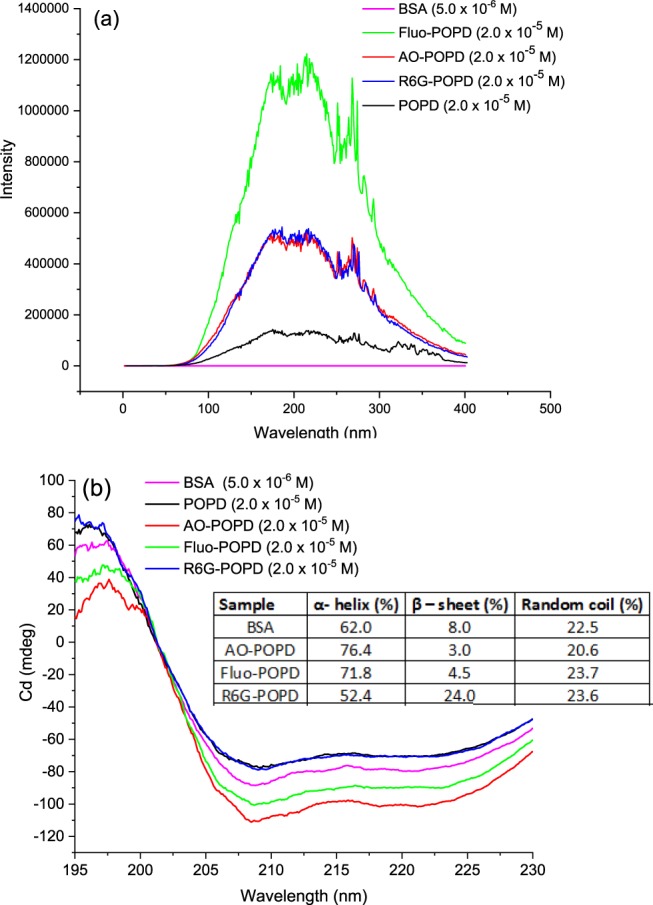
(**a**) RLS spectra of POPD and dye-POPD adducts, (**b**) Circular dichroism spectra of POPD and dye-POPD adducts.

RLS scattering gives information about aggregate formation in solutions which is indicated by the evolution of a characteristic peak in the fluorescence spectra and the intensity indicates the dimensions of the aggregate. The RLS signal of BSA solution, Fig. 5(a), indicated that BSA molecules did not undergo aggregation. In presence of POPD, BSA showed RLS signal of medium intensity revealing two peaks of same intensity lying on both sides of 200 nm indicating formation of small size aggregates. The RLS signal intensity of AO-POPD-BSA and R6G-POPD-BSA adducts showed much higher intensities as compared to the previous case. The two peaks of same intensity appeared on both sides of 200 nm. Formation of aggregates of larger size in these adducts were therefore confirmed. RLS signal intensity of Fluo-POPD-BSA adduct was far higher than the preceding two adducts revealing the formation of larger size aggregates. Hence, aggregation of BSA upon interaction with POPD and dye modified POPDs was confirmed. Circular dichroism is also used to monitor the changes in secondary and tertiary structure of BSA consequent to its interaction with different moieties that bring about changes in its conformation. The CD spectra of BSA usually show two negative bands in the UV region at 208 nm and 222 nm which are characteristic of the α-helix of protein^30^. CD results are presented in terms of mean residual ellipsicity (MRE) in deg cm^2^ dmol^−1^ as per the equation:

41 $${\rm{MRE}}={\rm{Observed}}\,{\rm{CD}}\,({\rm{mdeg}})/{{\rm{C}}}_{{\rm{p}}}{\rm{.}}\,{\rm{n}}{\rm{.}}\,{\rm{l}}.10$$

where C_p_ is the molar concentration of protein, n is the number of amino acid residues, 582 in BSA, and l is the path length^31^. The α-helical content of free BSA is calculated from MRE values at 208 nm using the equation:

42 $${\rm{\alpha }} \mbox{-} \mathrm{helix}\,( \% )=[(\,-{{\rm{MRE}}}_{{\rm{208}}}\,-\mathrm{4000})/(\mathrm{33},\,\mathrm{000}\mbox{--}\mathrm{4000})]\times {\rm{100}}$$

where MRE_208_ is the observed MRE value at 208 nm, 4000 is the MRE of the β–sheet and random coil conformation cross at 208 nm, and 33,000 is the MRE value of pure α-helix at 208 nm^31^. The CD spectra of POPD-BSA and R6G-PD-BSA, Fig. 5(b), were found to be of higher intensity than pure BSA, while those of AO-POPD-BSA and Fluo-POPD-BSA were observed to be of lower intensity than pure BSA which clearly indicated changes in α-helix and β-sheet content in the two cases. The α-helix content was observed to decrease in the former case, while it was noticed to increase in the later case. The CD spectra of BSA, POPD-BSA, AO-POPD-BSA, Fluo-POPD-BSA, and R6G-POPD-BSA, Fig. 5(b), showed two negative peaks at 208 nm and 222 nm. The MRE values of different moieties at 208 nm and α-helicity of BSA and BSA-polymer adducts were calculated. The values were found to decrease from 62% in free BSA to 52.4% in POPD/POPD-R6G–BSA, Fig. 5(b). The adducts AO-POPD-BSA and Fluo-POPD-BSA revealed α-helix values of 76.4% and 71.8% respectively. Furthermore, the CD spectrum of the protein in presence and absence of POPD and dye modified POPDs was observed to be of similar shape, indicating that the structure of BSA was predominantly α-helical even after binding with the polymers^32,33^. The α-helix content was found to be 52.4% in POPD-BSA and R6G-POPD-BSA adducts which was lower than the helical content of BSA (62%). Since the functional groups present in R6G-POPD and POPD have higher hydrophobic content, their molecules exist as coils in aqueous phase so as to minimize energy and penetrate into the polypeptide backbone of tryptophan-213. This has been reported by several authors^33,34^. The hydrophobic functional groups of R6G breakdown some of hydrogen bonds present in the secondary structure of the protein and reduce its α-helical content. Since R6G has more hydrophobic functional groups, R6G-POPD coils are larger than those of POPD. The presence of large coils increases the hydrophobic milieu around tryptophan-213 which enhances the fluorescence intensity around this amino acid residue. Fluo-POPD-BSA showed a notable increase of 9.8% in α-helical content (the total value being 71.8%). The fluorescein part of Fluo-POPD has hydrophilic groups of OH and COOH which prevents coiling of the molecules and therefore shows an extended structure. The hydrophilic groups present in Fluo dye penetrate the BSA molecule and cause the β–sheet to turn into α–helical coils, as reported by other authors^34^. This results in the formation of aggregates. The RLS scattering profile of the polymer also confirmed formation of large size aggregates. In this case, aqueous milieu is enhanced around tryptophan-213 which causes a decrease in the fluorescence intensity. The modified polymer AO-POPD has SO_3_^−^ and OH groups which hinder its coiling and therefore the molecules of this polymer reveal extended conformation in aqueous BSA solution and penetrate into polypeptide backbone of tryptophan-213 thereby increasing the α-helical content substantially up to 76.4%. This phenomena takes place due to folding of β–sheet into α–helix through formation of new hydrogen bonds. Hence, AO-POPD-BSA molecules form fairly large aggregates. The RLS scattering profile of this polymer also revealed large adduct formation, but smaller as compared to the Fluo-POPD-BSA. The 3-D spectra are useful in interpreting the structural changes in BSA upon interaction with POPD and dye doped POPDs. In this method excitation and emission wave lengths were scanned simultaneously with an increment of 10 nm and fluorescence intensity was measured at each step (Given in Supplementary Information, Fig. S12(a–d)). The excitation peak at 230 nm was correlated to the existence of π → π* of C=O associated with the polypeptide backbone of BSA. The changes in the fluorescence intensity at 230 nm excitation/340 nm emission couple were associated with changes in the helical structure of BSA and hydrophobic milieu of tryptophan and tyrosine amino acid residues^34^. Table 3 gives the fluorescence intensity of BSA (5 × 10^−6^ M), POPD-BSA, AO-POPD-BSA, Fluo-POPD-BSA and R6G-POPD-BSA, (2 × 10^−5^ M). AO-POPD-BSA revealed reduction in the fluorescence intensity of the peak at 230 nm excitation/340 nm emissions by 4.4% with respect to BSA. This behavior was attributed to the change in helical content of BSA which was also observed in CD studies. Likewise, Fluo-POPD-BSA showed 4.4% reduction in the fluorescence intensity of the above excitation/emission couple which was correlated to the enhancement in the α–helical content as revealed by CD spectral studies. Around 6.25% and 2.9% reduction in the fluorescence intensities of 280 nm excitation/340 nm emission couple was observed in case of AO-POPD–BSA and Fluo-POPD-BSA respectively. The results showed enhancement in polarity and aqueous milieu in the cavity around the tryptophan-213 reduced its hydrophobicity and consequently the fluorescence intensity. A reverse case of uncoiling of α–helix was observed in case of POPD-BSA and R6G-POPD-BSA adducts. The fluorescence intensity at 230 nm excitation and 340 emission was found to increase by 9.37% and 17.6% in POPD-BSA and R6G-POPD-BSA adducts respectively. The enhancement in fluorescence intensity was correlated to uncoiling of the α–helix of polypeptide backbone of domain II. This leads to partial destruction of the secondary structure of BSA through breaking of the hydrogen bonds present in the α–helix. The enhancement in fluorescence was intriguing in these cases. Due to the hydrophobic nature of the doped polymers, the hydrophobic character of the micro pockets around tryptophan-213 was enhanced that resulted into augmentation of its fluorescence intensity at 280 nm excitation and 340 nm emission.

**Table 3:** 3D fluorescence intensity of samples at 230, 280 nm excitation and 350 nm emission wavelengths.

Sample	Excitation	Emission	Intensity
BSA	230	350	340
	280	350	160
POPD-BSA	230	350	350
	280	350	170
AO-POPD-BSA	230	350	325
	280	350	150
Fluo-POPD-BSA	230	350	325
	280	350	155
R6G-POPD-BSA	230	350	400
	280	350	175

### DPV response of fluo-POPD and R6G-POPD towards BSA in PBS solution

**Figure 6:**
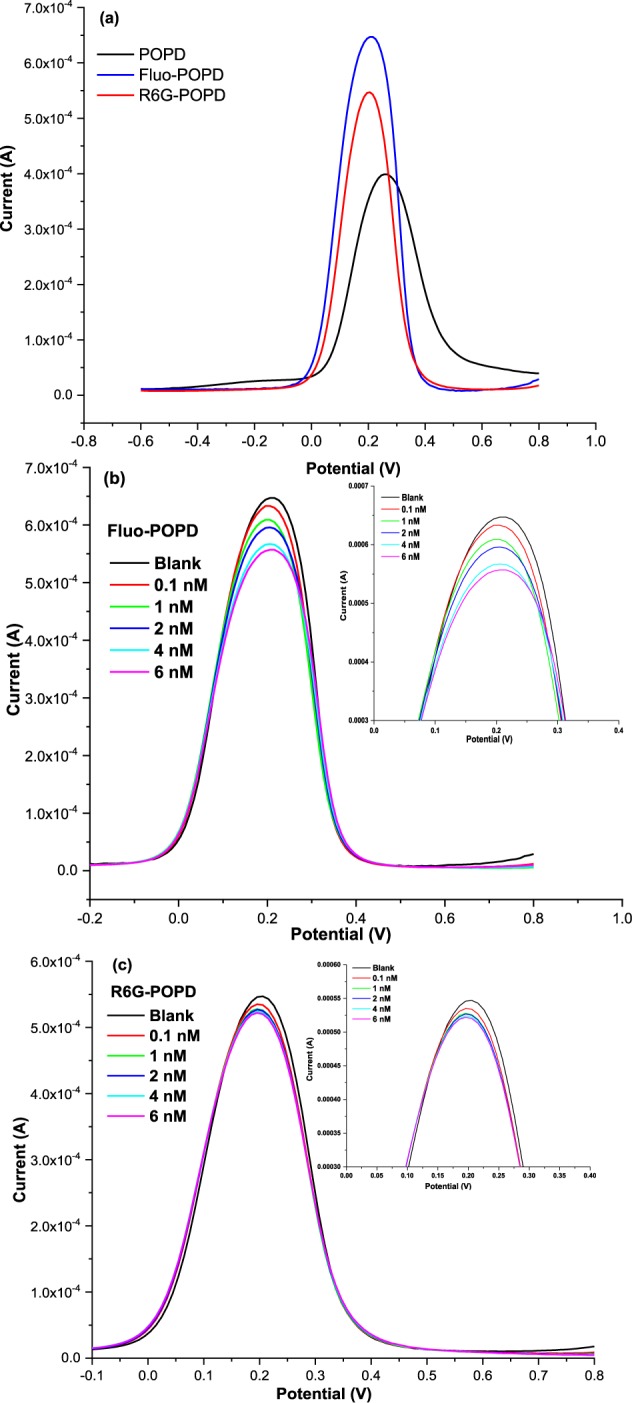
DPV response of different electrodes in detection buffer (BSA-PBS solution, pH 7.4 containing 5 mM [Fe(CN)_6_]^3−/4−^) (**a**) POPD and dye doped POPD adducts, (**b**) Fluo-POPD and (**c**) R6G-POPD (inset shows the enlarged image of the plot).

Electrochemical response studies were carried out using differential pulse voltammetry (DPV) technique. On comparing the DPV for POPD and dyes doped POPD in Fig. 6(a), the results revealed highest peak current for Fluo-POPD (6.4 × 10^−4^ A) followed by R6G-POPD (5.4 × 10^−4^ A), and POPD (3.9 × 10^−4^ A). When Fluo-POPD and R6G-POPD were deposited on the surface of the electrode, the current responses were much larger than pure POPD. The results can mainly be attributed to the synergistic effect of POPD with Fluorescein and R6G dyes. POPD, was able to electro-catalyze the dyes, possibly due to large surface area on electrode surface which provides active sites that facilitates the movement of charge and results in increase of current than pure POPD^35–37^.

BSA was used as target moiety in 0.05 M PBS, pH 7.4, for detection by POPD and dyes- doped POPD. BSA easily gets bound with the POPD that have partially positive charged amino group of POPD. There may be two different explanations for the interaction of BSA with POPD and dyes- doped POPD; first one is the formation of an electrochemically active compound with changes of the electrochemical parameters, second is the formation of electro-inactive complex without the changes of the electrochemical parameters^36,37^. The detection limit of BSA was investigated from 0.1 nM to 6 nM. A decrease in current was observed with increasing concentrations of BSA in Fluo-POPD Fig. 6(b) and R6G-POPD Fig. 6(c) due to the interaction of BSA with R6G-POPD and Fluo-POPD, which results in the formation of electro-inactive complex. The decrease in the concentration of R6G-POPD-and Fluo- POPD results in decrease of oxidation peak current. Fluo-POPD and R6G-POPD show decrease in current up to 6 nM after that no significant change in current was observed. That is the maximum limit of BSA reacting with the complex. The lower detection limit (LOD) is calculated to be as 1.35 nM for Fluo-POPD and 1.65 nM for R6G-POPD-R6G, using the equation:

43 $${\rm{LOD}}=({\rm{3}}\times {\rm{\sigma }})/{\rm{m}}$$

where σ is the standard deviation (SD) and m is the slope of the curve between current (A) and concentration of BSA (nM) in the linearity range of (0.1–6) nM and (0.1–6) nM for Fluo-POPD and R6G- POPD respectively. The value of the binding constant for the interaction of POPD and dyes- doped POPD with BSA can be determined by using Eq. (4.3). The equation was modified slightly according to the nature of the immobilized POPD and dyes- doped POPD with BSA

44 $$\mathrm{log}({\rm{1}}/[{\rm{BSA}}])=\,\mathrm{log}({\rm{K}})+\,\mathrm{log}({{\rm{I}}}_{{\rm{BSA}}\mbox{--}{\rm{POPD}}}/{{\rm{I}}}_{{\rm{POPD}}}-{{\rm{I}}}_{{\rm{BSA}}\mbox{--}{\rm{PAP}}})$$

where K is the apparent binding constant, I_POPD_ is the peak current of the POPD immobilized on ITO, and I_BSA–PAP_ is the peak current of the POPD in the presence of BSA–POPD complex after interaction of the BSA with the immobilized POPD. According to Eq. 4.3, a plot of log(1/[BSA]) against log(I_BSA–PAP_/(I_POPD_ − I_BSA–PAP_)) will give us the value for binding constant, K from the intercept of the linear plot. The calculated binding constant (K) for POPD was found to be 3.28 × 10^6^ L/mol. Similarly, K value for Fluo-POPD, R6G-POPD were found to be 3.98 × 10^6^ L/mol and 5.27 × 10^2^ L/mol.

### Morphological analysis of POPD and dye-POPD adducts

**Figure 7:**
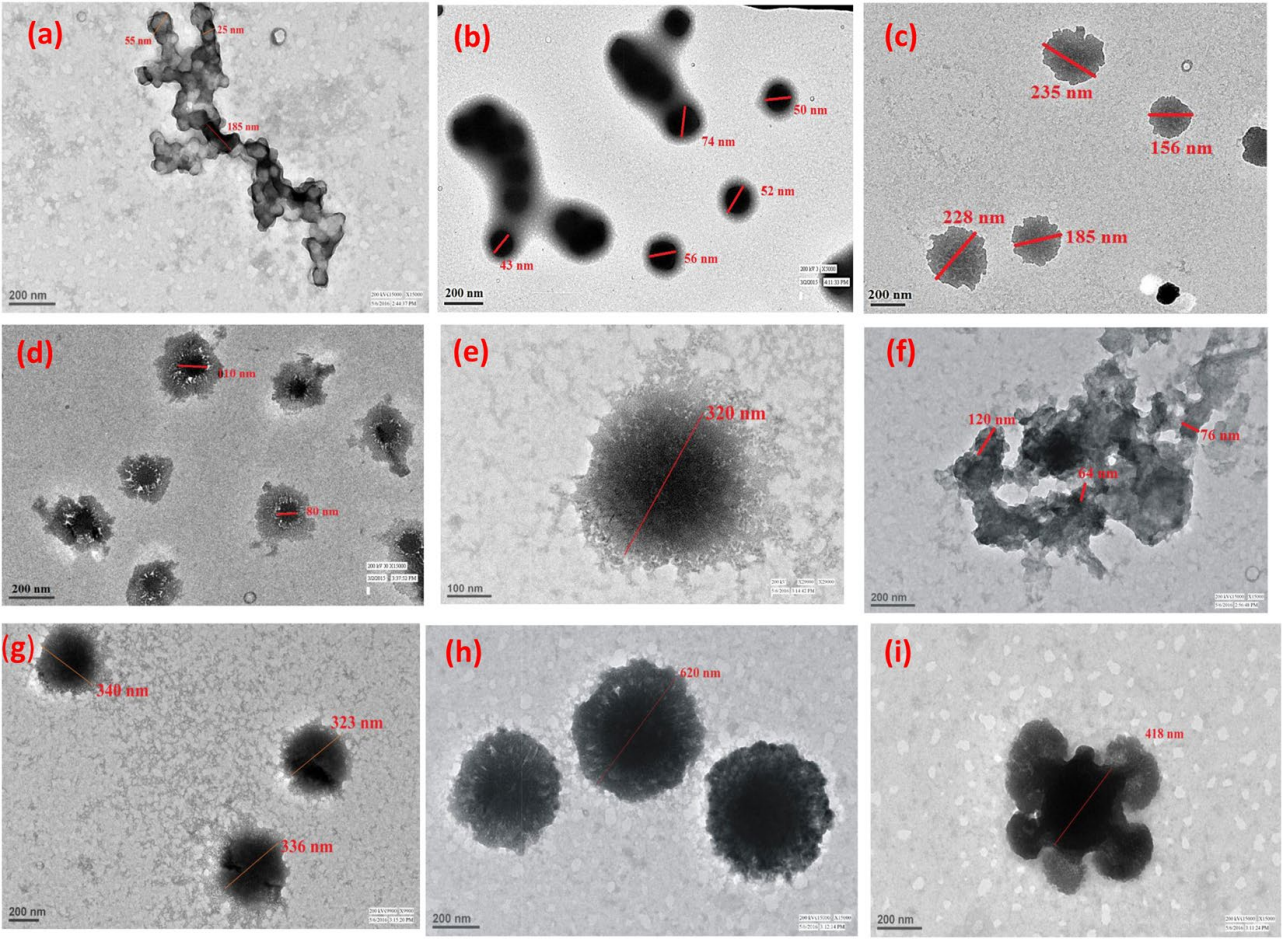
TEM images of (**a**) POPD, (**b**) AO-POPD (**c**) Fluo-POPD, (**d**) R6G-POPD, (**e**) BSA, (**f**) POPD- BSA, (**g**) AO-POPD-BSA, (**h**) Fluo-POPD-BSA, (**i**) R6G-POPD-BSA.

The TEM of POPD, Fig. 7(a), revealed the formation of chain like structure consisting of fused distorted spherical particles 40–50 nm. The morphology of AO-POPD, Fig. 7(b), exhibited dense spherical particles of POPD enclosed in a thin film of AO dye. The spherical particle sizes were observed to be ranging between 50 nm–70 nm and the morphology could be correlated to the formation of core-shell type structure. The TEM of Fluo-POPD, Fig. 7(c), revealed the formation of distorted spherical particles scattered uniformly with particle size ranging between 280–350 nm, while the TEM of R6G-POPD, Fig. 7(d), showed core-shell morphology composed of dense irregularly shaped spherical particles of POPD surrounded by a thin fragile core of dye molecules. The size of the core particles was found to be ranging between 80 nm–110 nm. The TEM of BSA revealed a fibrous spherical morphology, Fig. 7(e) and the size of the spherical particle was found to be 320 nm. The TEM of POPD-BSA adduct, Fig. 7(f), showed aggregated morphology forming large clusters of distorted particles. The CD studies also confirmed the formation of an expanded structure. The TEM of AO-POPD-BSA adduct, Fig. 7(g), showed formation of spherical particles as observed in the TEM of pure BSA but the particles were observed to have a dense core with size ranging between 300 nm–350 nm. The TEM of Fluo-POPD-BSA adduct Fig. 7(h), revealed the formation of dense granular spheres with size ranging between 600–620 nm while the TEM of R6G-POPD-BSA adduct, Fig. 7(i), exhibited a fused morphology composed of a dense sphere surrounded by hollow and distorted spherical clusters. The morphology obtained from the TEM analysis was well correlatesd with the results obtained from CD and RLS studies.

### Confocal microscopy of POPD and dye-POPD adducts

**Figure 8:**
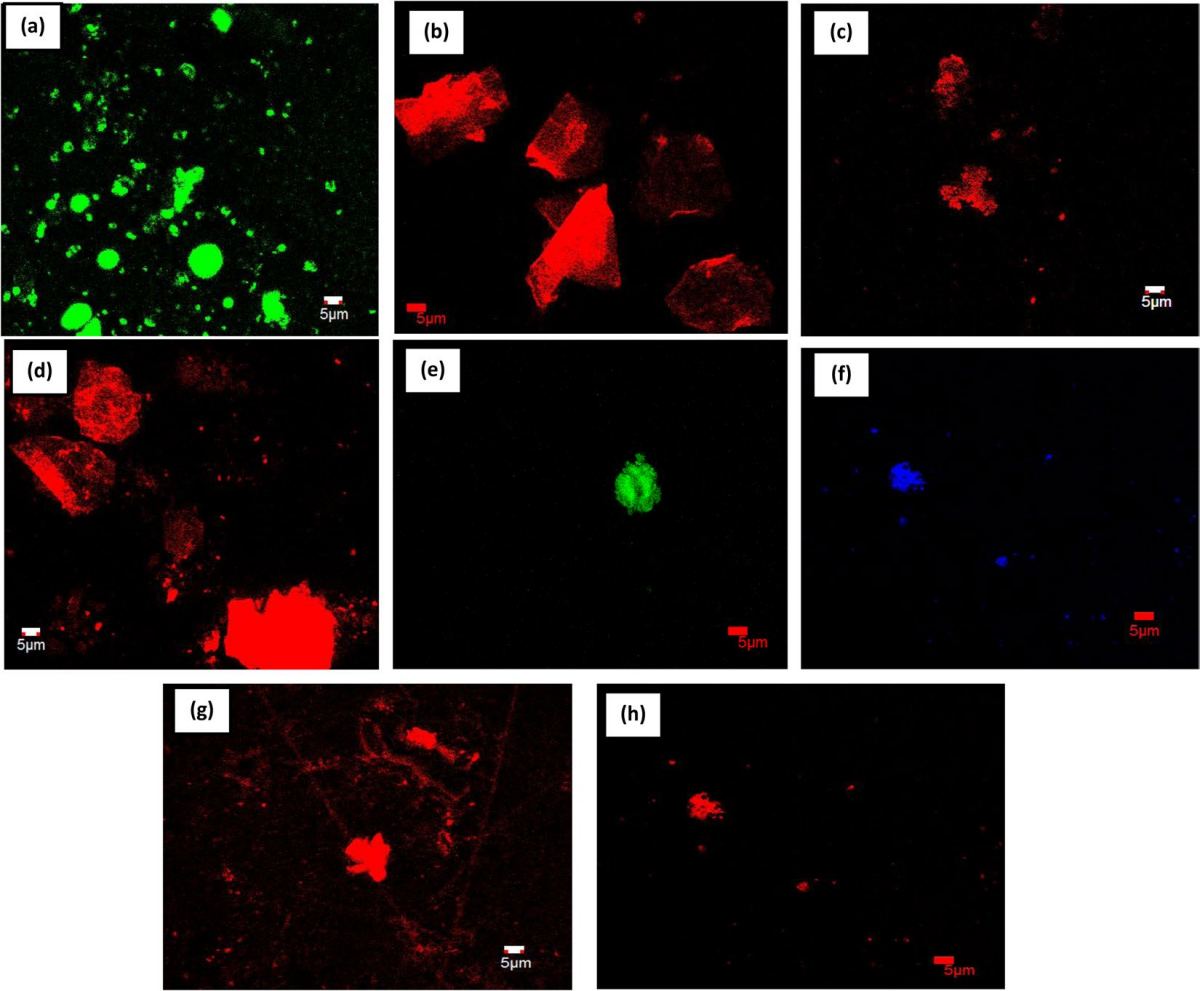
Confocal images of (**a**) POPD, (**b**) AO-POPD (**c**) Fluo-POPD, (**d**) R6G-POPD, (**e**) POPD-BSA, (**f**) AO-POPD-BSA, (**g**) Fluo-POPD-BSA, (**h**) R6G-POPD-BSA.

The confocal micrograph of POPD, Fig. 8(a), revealed bright and intense green emission from particles having distorted spherical morphology, while the confocal micrograph of AO-POPD Fig. 8(b), showed intense red emission from irregularly shaped aggregated structures. The confocal micrographs of Fluo-POPD, Fig. 8(c), and R6G-POPD, Fig. 8(d), revealed bright red emission from distorted spherical particles forming huge clusters in the later case. Confocal microscopy of POPD and dye doped POPDs clearly showed the modification of POPD by dyes through different coloured micrographs. The confocal micrograph of POPD-BSA adduct, Fig. 8(e), revealed green emission matching with its fluorescence spectra and the fluorescing spherical particle of the polymer appeared to be discrete. The confocal micrograph of AO-POPD-BSA adduct, Fig. 8(f), revealed blue emission from discrete particles showing distorted spherical morphology. The blue emission could be attributed to strong binding with the BSA causing Stokes shift which permitted only blue emission. However, Fluo-POPD-BSA and R6G-POPD-BSA adducts, Fig. 8(g,h), revealed discrete red colored particles which appeared to be widely distributed. The binding of POPD and dye modified POPDs resulted from the interaction of the amino/imino groups especially with tryptophan-213 of BSA forming a ground state intermolecular complex.

Hence, the modified polymers-BSA adducts clearly showed binding of POPD and dye doped POPDs through variation in emission observed in confocal microscopy.

### Live cell imaging using POPD and dye-POPD adducts as markers

**Figure 9:**
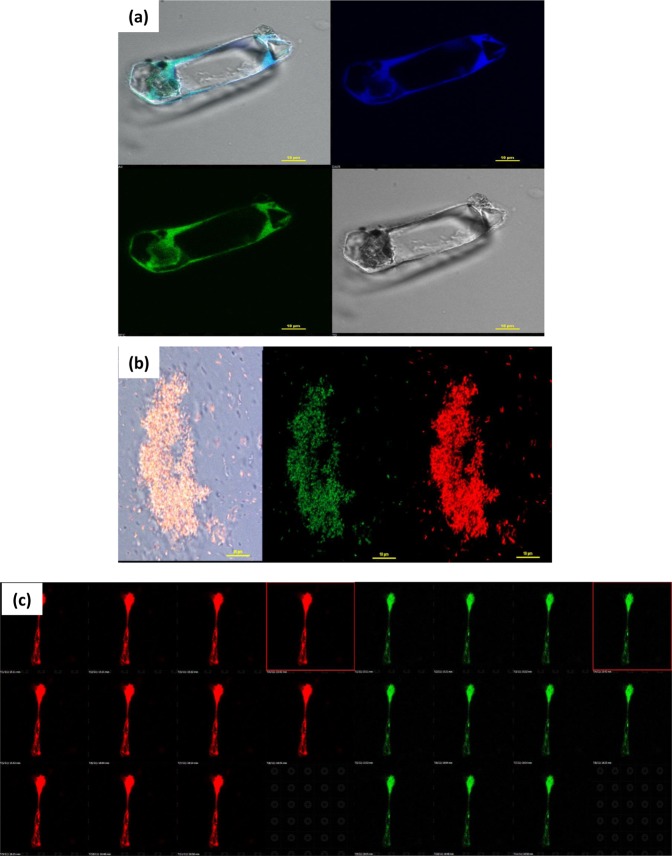
Live cell images of (**a**) POPD with *E*. *coli*, (**b**) POPD-R6G with *E*. *coli*, (**c**) Serial optical sections of *E*. *coli* with POPD-R6G.

Since POPD-BSA and POPD-R6G-BSA showed small and medium size aggregates (cf. RLS Scattering) having highest quantum yield and molar extinction coefficient among the polymers under investigation, these polymers were chosen for targeting bacteria. *E*. *coli* as used as model bacteria to explore the efficiency of POPD and POPD-R6G as fluorescent markers. The live *E*. *coli* bacteria cells were interacted with POPD and POPD-R6G as given in the experimental section and were viewed under the live cell imaging microscope. The live cell images of POPD and POPD-R6G, Fig. 9(a–c) revealed that these could be used to label the bacteria to perform biological imaging and analysis. The live cell imaging of POPD labelled *E*. *coli*, Fig. 9(a), clearly showed that POPD particles were bound to the outer membrane and the cyotoplasm in the *E*. *coli* cells^38^. The green channel revealed a brighter outer membrane wall than the blue channel. POPD stained *E*. *coli* showed bright green fluorescence originating from hydrogen bonded POPD to the bacterial cell wall. It appeared that the POPD particles were unable to target the cytoplasmic molecules and seemed to only label the membrane wall. This is because POPD particles are unable to overcome the hydrogen bond forces to penetrate into cytoplasmic region^38^. For POPD-R6G, a huge live bacterial cluster was imaged, Fig. 9(b). It was observed that the nanoparticles of R6G-POPD deeply penetrated the cell wall and successfully stained the cytoplasm as well as nucleus of bacteria. The probe appeared to penetrate the bacterial cell walls through endocytic process. Z-stack of POPD-R6G labelled *E*. *coli*, Fig. 9(c), clearly revealed the penetration of these nano particles into the cytoplasm of the bacteria. This shows that R6G-POPD is bio-compatible. Since this polymer is soluble in water by itself, it is easier to use it in bioimaging. It was found to bleach in acquisition time of 14 minutes in laser illumination (Activation and excitation laser wavelengths and intensities were 405 nm at 0.06 kW cm^−2^ and 561 nm at 5 kW cm^−2^. Images were acquired with a frame rate of 30 frames s^–1^. Total acquisition time for Fig. 9(c) was ~14 min, (Video given in supplementary information). R6G-POPD is a simple structured, uncluttered fluorescent biomarker and can detect both BSA and *E*. *coli* bacteria. Dense and brighter labeling of cytoplasm and nucleic acid was achieved in the red channel as compared to the green channel.

## Conclusion

POPD and dye-POPD adducts were successfully synthesized using AO, Fluo and R6G dyes. Doping was established by UV-visible and XPS studies through hydrogen bonding between NH group of the polymer and C=O/SO^−^, S=O group of the dyes. The spectrophotometric determination of hydrogen bonding was also carried out by the addition of urea and sodium chloride to dye-POPD adducts. The λ_emis_ values of these adducts showed low Stoke’s shift in the xanthene dyes and a high Stoke’s shift in the azo dye. AO-POPD revealed the formation of a strained structure, while the structure of Fluo-POPD was noticed to be least strained. Intrinsic fluorescence decay constant (k^0^_f_) values were observed to be 0.071, 0.072, 0.153 and 0.172 (10 ^8^ s^−1^) for POPD, AO-POPD, Fluo-POPD and R6G-POPD respectively. The values were correlated to the increasing strain-free molecular structure of the adducts. The lowest detection limit of BSA by DPV studies was found to be 1.35 nM in presence of Fluo-POPD and and 1.65 nM in presence of R6G-POPD. The binding constant values of BSA were obtained as 3.98 × 10^6^ Lmol^−1^ and 5.27 × 10^2^ Lmol^−1^ in presence of Fluo-POPD and R6G-POPD respectively. POPD and dye-POPD adducts were found to quench the fluorescence of BSA through complex formation in the ground state and electron transfer in the excited state. CD studies revealed that POPD and R6G-POPD, made smaller aggregates with BSA and partially disrupted the helical structure of BSA, whereas AO-POPD and Fluo-POPD, which made larger aggregates with BSA, enhanced its helical content. POPD and R6G-POPD augmented the hydrophobicity in the micro-environment around amino acid residue-213, but AO-POPD and Fluo-POPD, increased aqueous milieu in the above micro-region. Live cell imaging of POPD interacted *E*. *coli* showed that POPD nano particles were bound to the outer membrane of *E*. *coli* while R6G-POPD was observed to deeply penetrate the cell wall and successfully stained the cytoplasm as well as nucleus of bacteria. Thus, doping of POPD could to utilized to design florescent biomarkers for imaging as well as detection of biological analytes and could be easily tuned to emit in the desired range by doping with different dyes.

## Supplementary information

Supplementary Information

## References

[1] Byrne SJ (2006). Optimisation of the synthesis and modification of CdTe quantum dots for enhanced live cell imaging. J. Mater. Chem..

[2] Chen M-L, Liu J-W, Hu B, Chen M-L, Wang J-H (2011). Conjugation of quantum dots with graphene for fluorescence imaging of live cells. Analyst.

[3] Li J, Zhu J-J (2013). Quantum dots for fluorescent biosensing and bio-imaging applications. Analyst.

[4] Jaiswal JK, Goldman ER, Mattoussi H, Simon SM (2004). Use of quantum dots for live cell imaging. Nat. Methods.

[5] Jin Y, Ye F, Zeigler M, Wu C, Chiu DT (2011). Near-infrared fluorescent dye-doped semiconducting polymer dots. ACS Nano.

[6] Feng L (2013). Conjugated polymer nanoparticles: preparation, properties, functionalization and biological applications. Chem. Soc. Rev..

[7] Bhattacharyya S, Prashanthi S, Bangal PR, Patra A (2013). Photophysics and dynamics of dye-doped conjugated polymer nanoparticles by time-resolved and fluorescence correlation spectroscopy. J. Phys. Chem. C.

[8] Zhao S (2017). Portable solid rapid quantitative detection for Cu2+ ions: Tuning the detection range limits of fluorescent conducting polymer dots. J. Mater. Res..

[9] Song X, Sun H, Yang S, Zhao S, Liao F (2016). Synthesis of photoluminescent o-phenylenediamine–m-phenylenediamine copolymer nanospheres: An effective fluorescent sensing platform for selective and sensitive detection of chromium(VI) ion *J*. Lumines..

[10] Zhao S (2016). Polydopamine dots as an ultrasensitive fluorescent probe switch for Cr(VI). in vitro, J. Appl. Polym. Sci..

[11] Feng L-J, Zhang X-H, Zhao D-M, Wang S-F (2011). Electrochemical studies of bovine serum albumin immobilization onto the poly-o-phenylenediamine and carbon-coated nickel composite film and its interaction with papaverine. Sens. Actuat. B, Chem..

[12] Jadoun S, Sharma V, Ashraf SM, Riaz U (2017). Sonolytic doping of poly (1-naphthylamine) with luminol: influence on spectral, morphological and fluorescent characteristics. Coll. Polym. Sci..

[13] Riaz U, Ashraf SM, Fatima T, Jadoun S (2017). Tuning the spectral, thermal and fluorescent properties of conjugated polymers via random copolymerization of hole transporting monomers. Spectrochim. Acta Part A: Mol. Biomol. Spectr..

[14] Liao F (2015). Photoinduced electron transfer of poly(o-phenylenediamine)–Rhodamine B copolymer dots: application in ultrasensitive detection of nitrite *in vivo*. J. Mater. Chem. A.

[15] Riaz U, Ashraf SM, Aleem S, Budhiraja V, Jadoun S (2016). Microwave-assisted green synthesis of some nanoconjugated copolymers: characterisation and fluorescence quenching studies with bovine serum albumin. New J. Chem..

[16] Fuenzalida JP (2014). Immobilization of Hydrophilic Low Molecular-Weight Molecules in Nanoparticles of Chitosan/Poly(sodium 4-styrenesulfonate) Assisted by Aromatic−Aromatic Interactions. J. Phys. Chem. B.

[17] Hamlin, J. D., Philps, D. A. S. & Whitting, A. UV/Visible spectroscopic studies of the e€ects of common salt and urea upon reactive dye solutions *Dyes and Pig*. **41**, 137–142 (1999).

[18] Huang Y, Zhang Z, Zhang D, Lv J (2001). Flow-injection analysis chemiluminescence detection combined with microdialysis sampling for studying protein binding of drug. Talanta.

[19] Liu J (2004). Spectrofluorimetric study of the binding of daphnetin to bovine serum albumin. J. Pharm. Biomed. Anal..

[20] Ross PD, Subramanian S (1981). Thermodynamics of protein association reactions: forces contributing to stability. Biochem..

[21] Khani O (2011). Synthesis and characterizations of ultra-small ZnS and Zn_(1−x)_Fe_x_S quantum dots in aqueous media and spectroscopic study of their interactions with bovine serum albumin. Spectrochim Acta, Part A.

[22] Riaz U, Ashraf SM, Kumar S, Zeeshan M, Jadoun S (2016). Microwave-assisted solid state intercalation of Rhodamine B and polycarbazole in bentonite clay interlayer space: structural characterization and photophysics of double intercalation. RSC Adv..

[23] Ethiraj AS (2005). Photoluminescent core-shell particles of organic dye in silica. J. Lumines..

[24] Villoslada IM (2010). Comparative Study of the Self-Aggregation of Rhodamine 6G in the Presence of Poly(sodium 4-styrenesulfonate), Poly(*N*-phenylmaleimide-*co*-acrylic acid), Poly(styrene-*alt*-maleic acid), and Poly(sodium acrylate),. J. Phys. Chem. B.

[25] Samanta S, Roy P, Kar P (2016). Structure and properties of conducting poly(o-phenylenediamine) synthesized in different inorganic acid medium. Macromol. Res..

[26] Stern O, Volmer M (1919). Uber die abklingungszeit der fluoreszenz. Phys Z.

[27] Jangid NK, Chauhan NPS, Punjabi PB (2015). Preparation and Characterization of Polyanilines Bearing Rhodamine 6-G and Azure B as Pendant Groups. J. Macromol. Chem. Part A@ Pure Appl. Chem..

[28] Zare EN, Lakouraj MM, Ghasemi S, Moosavi E (2015). Emulsion polymerization for the fabrication of poly(o-phenylenediamine)@multi-walled carbon nanotubes nanocomposites: characterization and their application in the corrosion protection of 316L SS. RSC Adv..

[29] Mariam J, Dongre PM, Kothari DC (2011). Study of interaction of silver nanoparticles with bovine serum albumin using fluorescence spectroscopy. J. Fluoresc..

[30] Zhang L-N, Wu F-Y, Liu A-H (2011). Study of the interaction between 2,5-di-[2-(4-hydroxy-phenyl)ethylene]-terephthalonitril and bovine serum albumin by fluorescence spectroscopy. Spectrochim. Acta A.

[31] Asha Jhonsi M, Kathiravan A, Renganathan R (2009). Spectroscopic studies on the interaction of colloidal capped CdS nanoparticles with bovine serum albumin. Colloids and Surfaces B: Biointerf..

[32] Tian Z (2015). Spectroscopic study on the interaction between mononaphthalimide spermidine (MINS) and bovine serum albumin (BSA). J. Photochem. Photobiol B Biol..

[33] He W (2006). Molecular modeling and spectroscopic studies on the binding of guaiacol to human serum albumin. J. Photochem. Photobiol. A.

[34] Sandhya B, Hegde AH, Kalanur SS, Katrahalli U (2011). Seetharamappa, The effect of anti-tubercular drug, ethionamide on the secondary structure of serum albumins: A biophysical study. J. Pharma. Biomed. Anal..

[35] Liu Z (2014). Simultaneous Determination of Orange G and Orange II in Industrial Wastewater by a Novel Fe_2_O_3_/MWCNTs-COOH/OP Modified Carbon Paste Electrode. Electrochimica Acta.

[36] Patel MK (2013). Nanostructured magnesium oxide biosensing platform for cholera detection. Appl. Phy. Lett..

[37] Ibrahim MS (2001). Voltammetric studies of the interaction of nogalamycin antitumor drug with DNA. Anal. Chim. Acta.

[38] Riaz U (2017). Influence of Luminol Doping of Poly(*o*-phenylenediamine) on the Spectral, Morphological, and Fluorescent properties: A Potential Fluorescent Marker for Early detection and Diagnosis of *Leishmania donovani*. ACS Appl. Mater. Interf..

