# Supplementary material for: Spectroscopic and Biophysical Interaction Studies of Water-soluble Dye modified poly(o-phenylenediamine) for its Potential Application in BSA Detection and Bioimaging
Source: Sci Rep. 2019 Jun 12;9:8544. doi: 10.1038/s41598-019-44910-z (PMC6561923; doi:10.1038/s41598-019-44910-z)
Supplement: Supplementary file 1 — Supplementary Information [file 41598_2019_44910_MOESM1_ESM.pdf]

Supplementary Information

Spectroscopic and Biophysical Interaction Studies of Water-soluble Dye modified poly(o-phenylenediamine) for its Potential Application in BSA Detection and Bioimaging

Ufana Riaz[a*], S.M.Ashraf[a†], Sapana Jadoun[a], Vaibhav Budhiraja[a], and Prabhat Kumar[b],

[a]Materials Research Laboratory Department of Chemistry, Jamia Millia Islamia,New Delhi-110025, India,

[b]Advanced Instrumentation Research Facility, Jawaharlal Nehru University, New Delhi- 110067

[*]corresponding author email: ufana2002@yahoo.co.in, [†] now retired

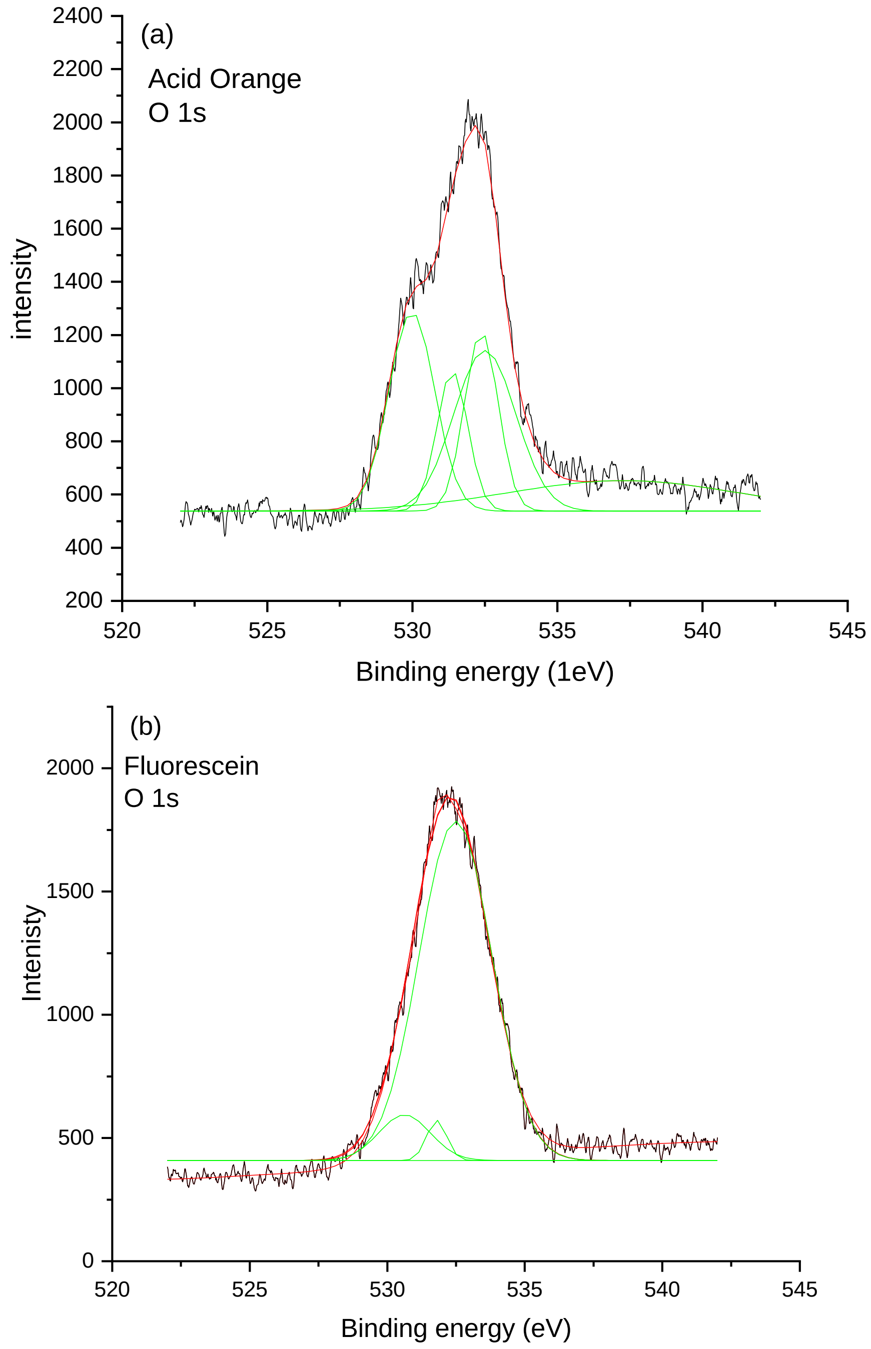

Figure S1 XPS spectra of (a) Acid orange (AO) dye (O 1s) , (b) Fluorescein (Fluo) dye (O 1s)

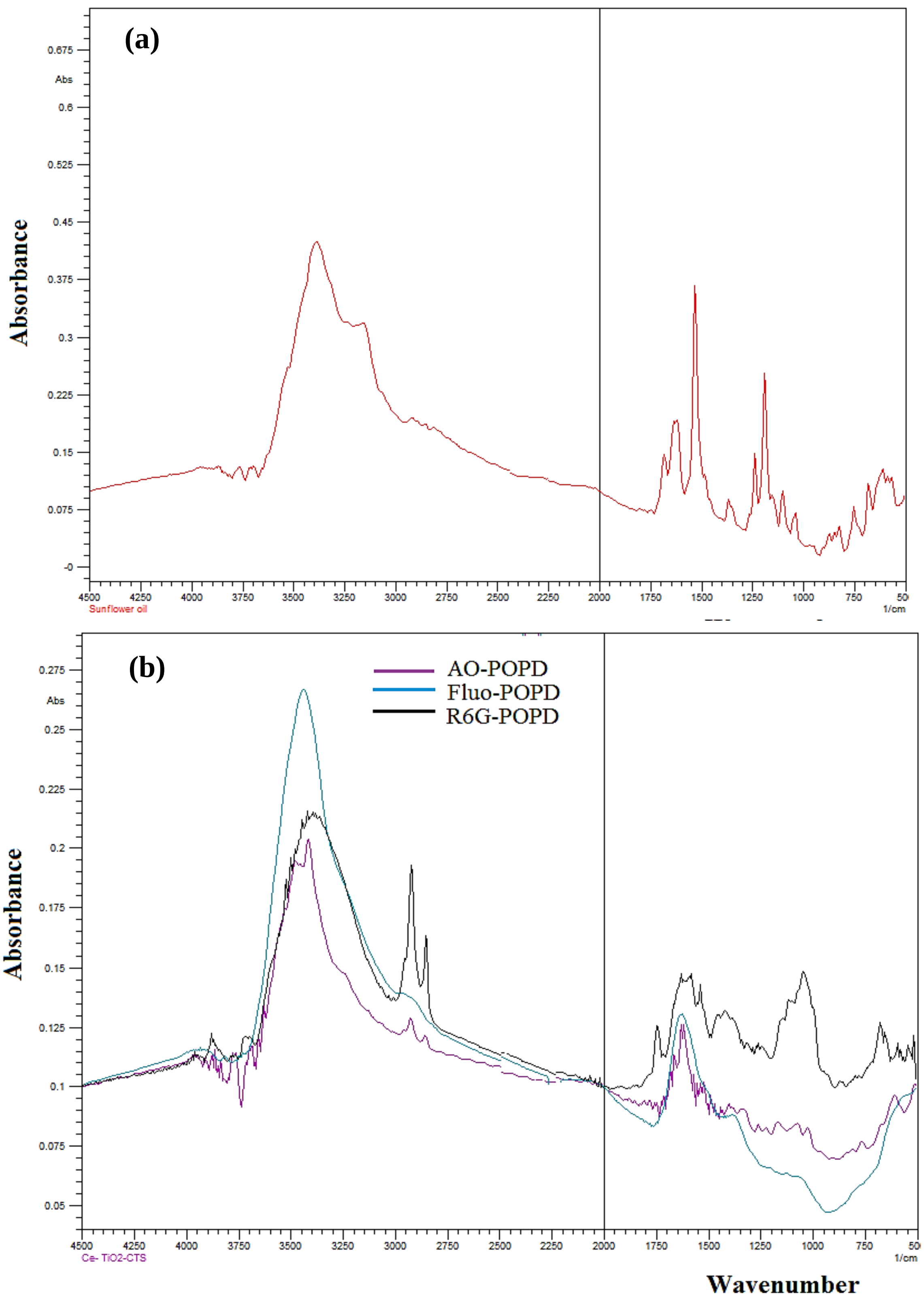

Figure S2 IR Spectra of (a) POPD and (b) dye modified POPD

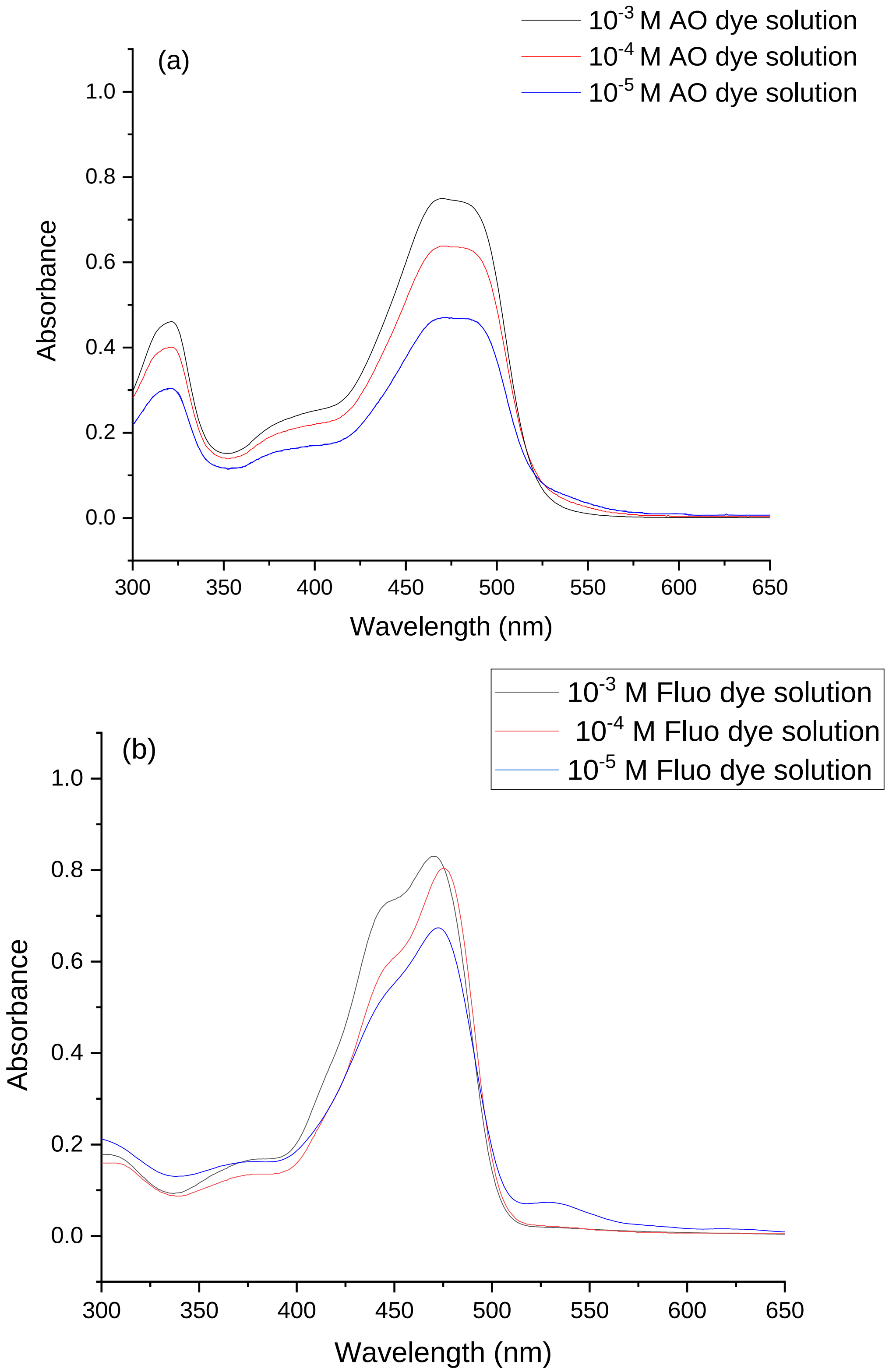

10-3 M AO dye solution
10-4 M AO dye solution
10-5 M AO dye solution
(a)
Absorbance
Wavelength (nm)
10-3 M Fluo dye solution
10-4 M Fluo dye solution
10-5 M Fluo dye solution
(b)
Absorbance
Wavelength (nm)

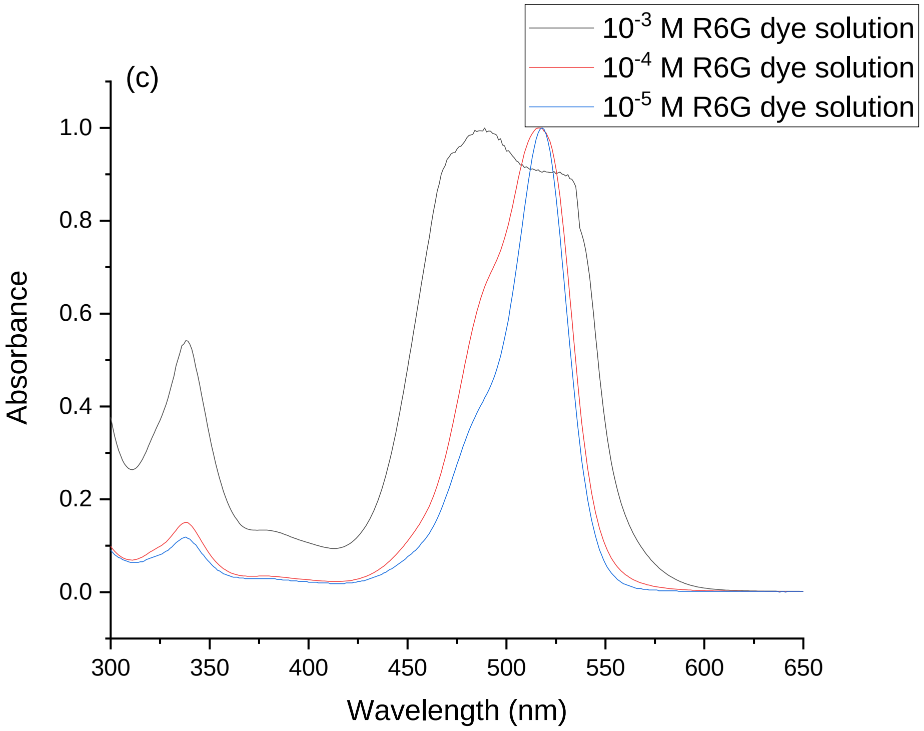

Figure S3 UV visible spectra of dye solutions of (a) AO dye, (b) Fluo dye and (c) R6G dye

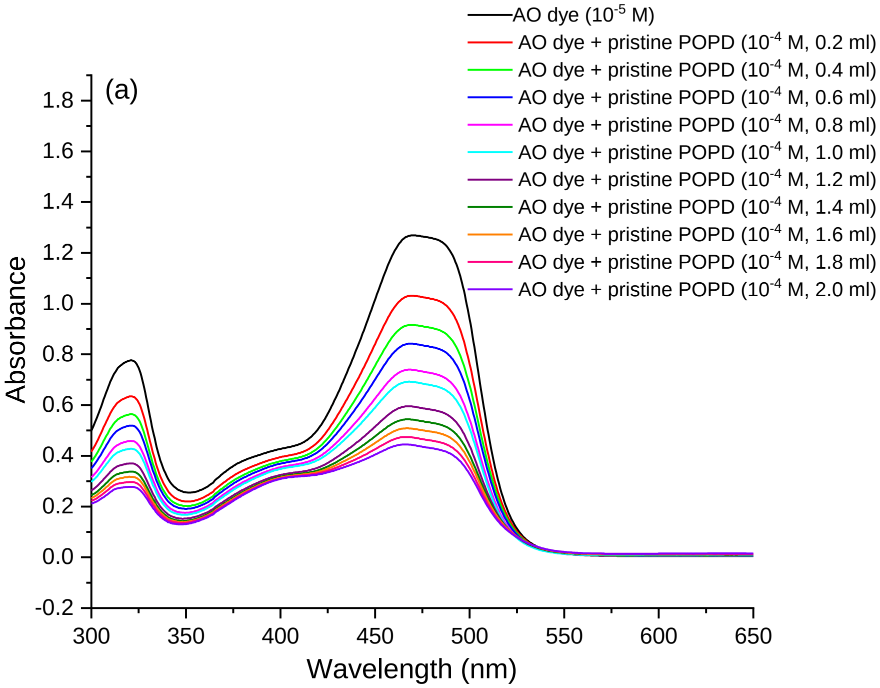

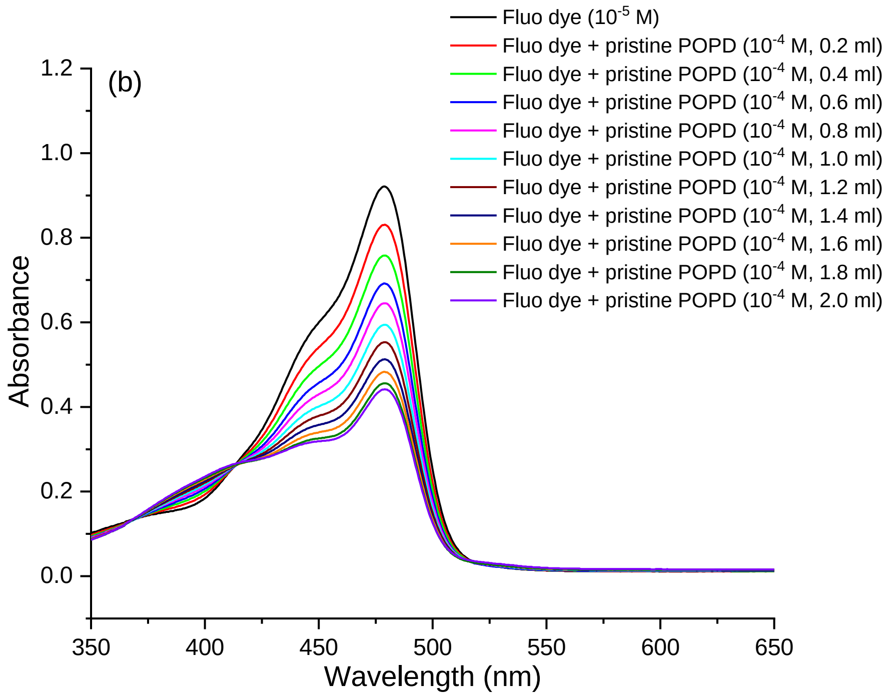
Fluo dye (10-5 M)
Fluo dye + pristine POPD (10-4 M, 0.2 ml)
Fluo dye + pristine POPD (10-4 M, 0.4 ml)
Fluo dye + pristine POPD (10-4 M, 0.6 ml)
Fluo dye + pristine POPD (10-4 M, 0.8 ml)
Fluo dye + pristine POPD (10-4 M, 1.0 ml)
Fluo dye + pristine POPD (10-4 M, 1.2 ml)
Fluo dye + pristine POPD (10-4 M, 1.4 ml)
Fluo dye + pristine POPD (10-4 M, 1.6 ml)
Fluo dye + pristine POPD (10-4 M, 1.8 ml)
Fluo dye + pristine POPD (10-4 M, 2.0 ml)
(b)
Absorbance
Wavelength (nm)
0.0
0.2
0.4
0.6
0.8
1.0
1.2
350
400
450
500
550
600
650

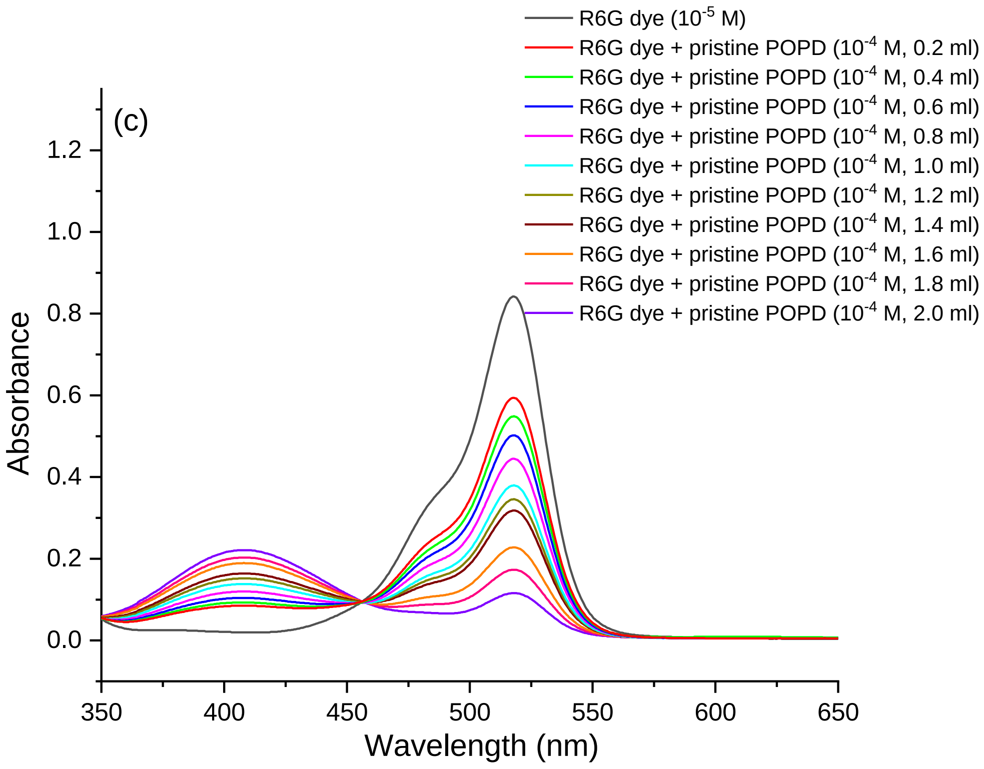
R6G dye (10-5 M)
R6G dye + pristine POPD (10-4 M, 0.2 ml)
R6G dye + pristine POPD (10-4 M, 0.4 ml)
R6G dye + pristine POPD (10-4 M, 0.6 ml)
R6G dye + pristine POPD (10-4 M, 0.8 ml)
R6G dye + pristine POPD (10-4 M, 1.0 ml)
R6G dye + pristine POPD (10-4 M, 1.2 ml)
R6G dye + pristine POPD (10-4 M, 1.4 ml)
R6G dye + pristine POPD (10-4 M, 1.6 ml)
R6G dye + pristine POPD (10-4 M, 1.8 ml)
R6G dye + pristine POPD (10-4 M, 2.0 ml)
(c)
Absorbance
Wavelength (nm)
0.0
0.2
0.4
0.6
0.8
1.0
1.2
350
400
450
500
550
600
650

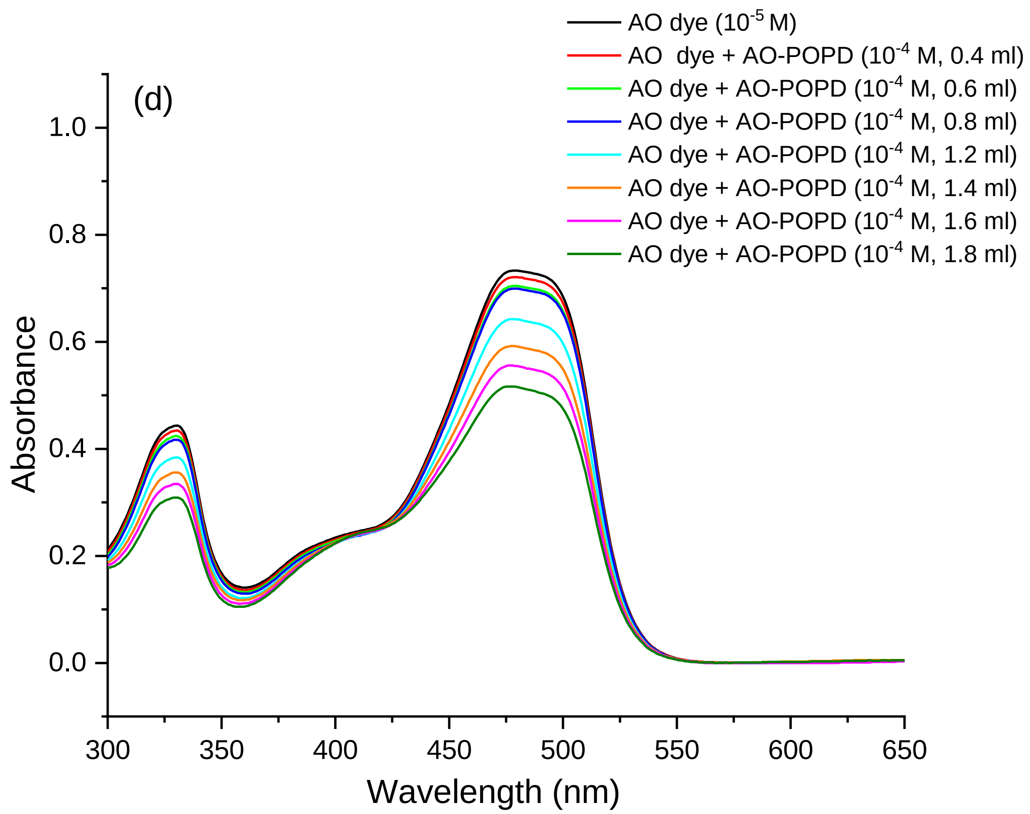
(d)
AO dye (10^{-5} M)
AO dye + AO-POPD (10^{-4} M, 0.4 ml)
AO dye + AO-POPD (10^{-4} M, 0.6 ml)
AO dye + AO-POPD (10^{-4} M, 0.8 ml)
AO dye + AO-POPD (10^{-4} M, 1.2 ml)
AO dye + AO-POPD (10^{-4} M, 1.4 ml)
AO dye + AO-POPD (10^{-4} M, 1.6 ml)
AO dye + AO-POPD (10^{-4} M, 1.8 ml)
Absorbance
Wavelength (nm)
0.0
0.2
0.4
0.6
0.8
1.0
300
350
400
450
500
550
600
650

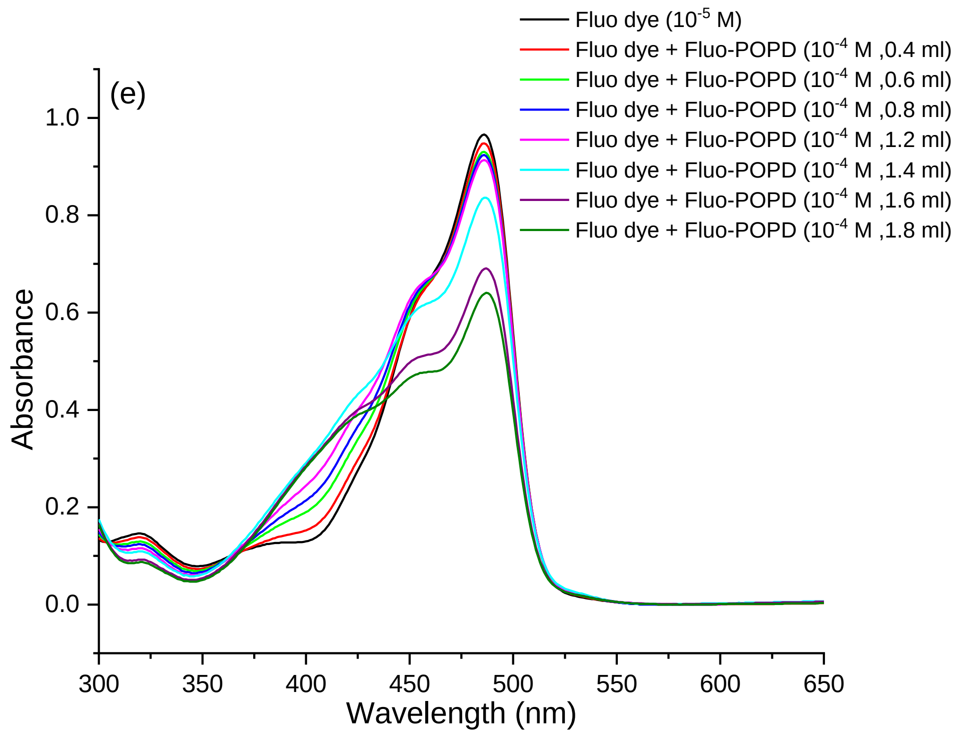
(e)
Fluo dye (10^{-5} M)
Fluo dye + Fluo-POPD (10^{-4} M ,0.4 ml)
Fluo dye + Fluo-POPD (10^{-4} M ,0.6 ml)
Fluo dye + Fluo-POPD (10^{-4} M ,0.8 ml)
Fluo dye + Fluo-POPD (10^{-4} M ,1.2 ml)
Fluo dye + Fluo-POPD (10^{-4} M ,1.4 ml)
Fluo dye + Fluo-POPD (10^{-4} M ,1.6 ml)
Fluo dye + Fluo-POPD (10^{-4} M ,1.8 ml)
Absorbance
Wavelength (nm)
0.0
0.2
0.4
0.6
0.8
1.0
300
350
400
450
500
550
600
650

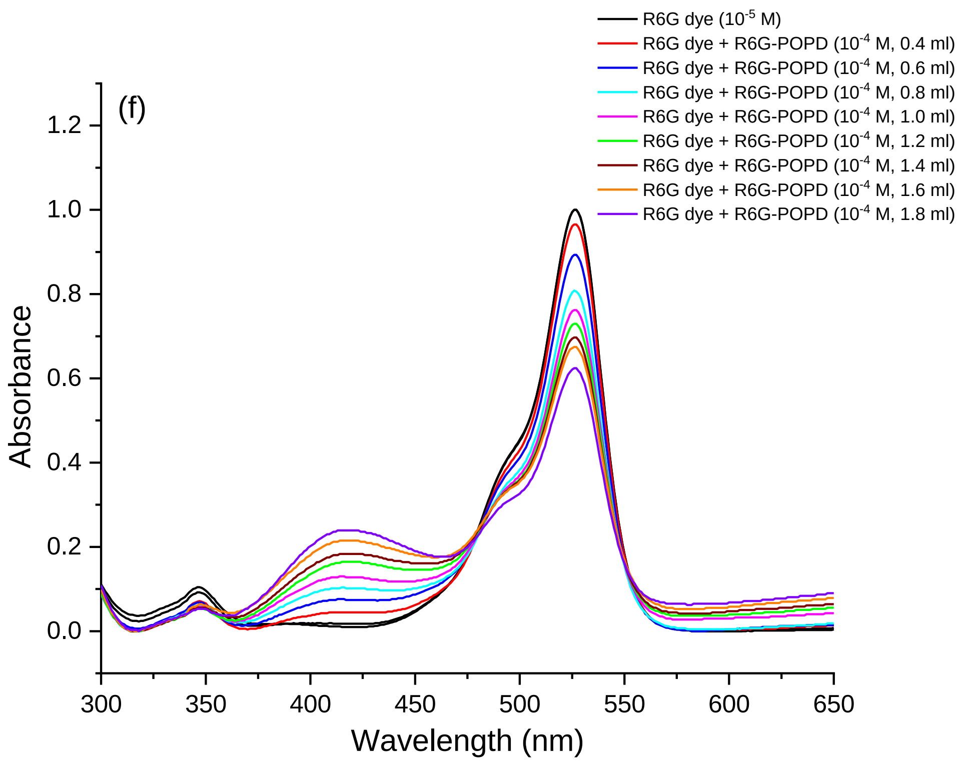

Figure S4 Effect of addition of POPD and dye doped POPDs to dye solutions

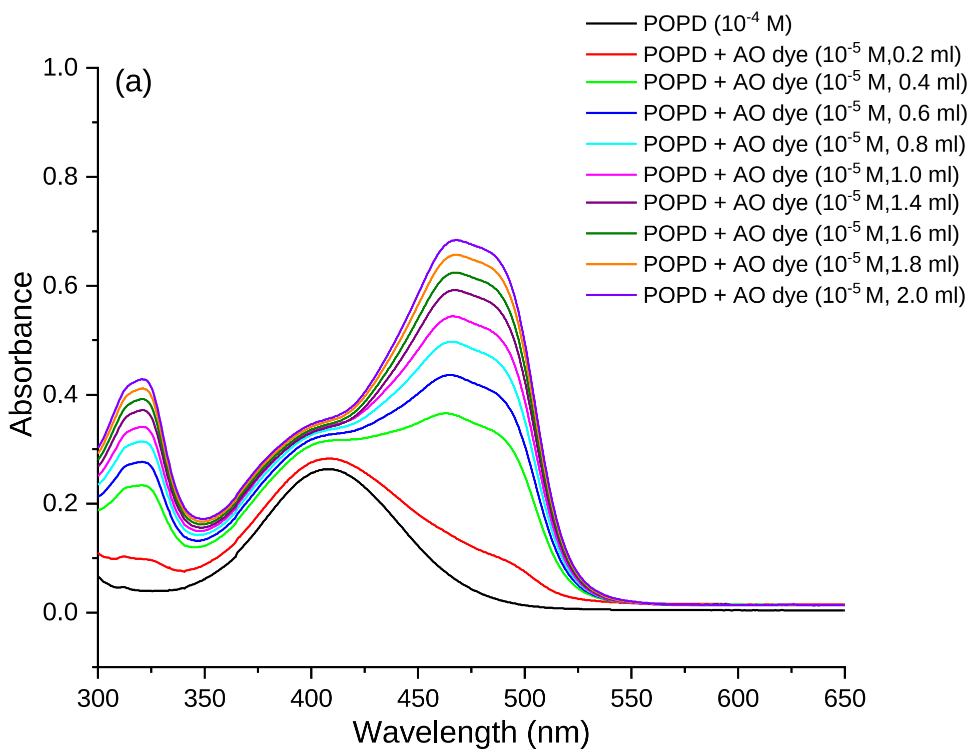
(a)
POPD (10^{-4} M)
POPD + AO dye (10^{-5} M,0.2 ml)
POPD + AO dye (10^{-5} M, 0.4 ml)
POPD + AO dye (10^{-5} M, 0.6 ml)
POPD + AO dye (10^{-5} M, 0.8 ml)
POPD + AO dye (10^{-5} M,1.0 ml)
POPD + AO dye (10^{-5} M,1.4 ml)
POPD + AO dye (10^{-5} M,1.6 ml)
POPD + AO dye (10^{-5} M,1.8 ml)
POPD + AO dye (10^{-5} M, 2.0 ml)
Absorbance
Wavelength (nm)
0.0
0.2
0.4
0.6
0.8
1.0
300
350
400
450
500
550
600
650

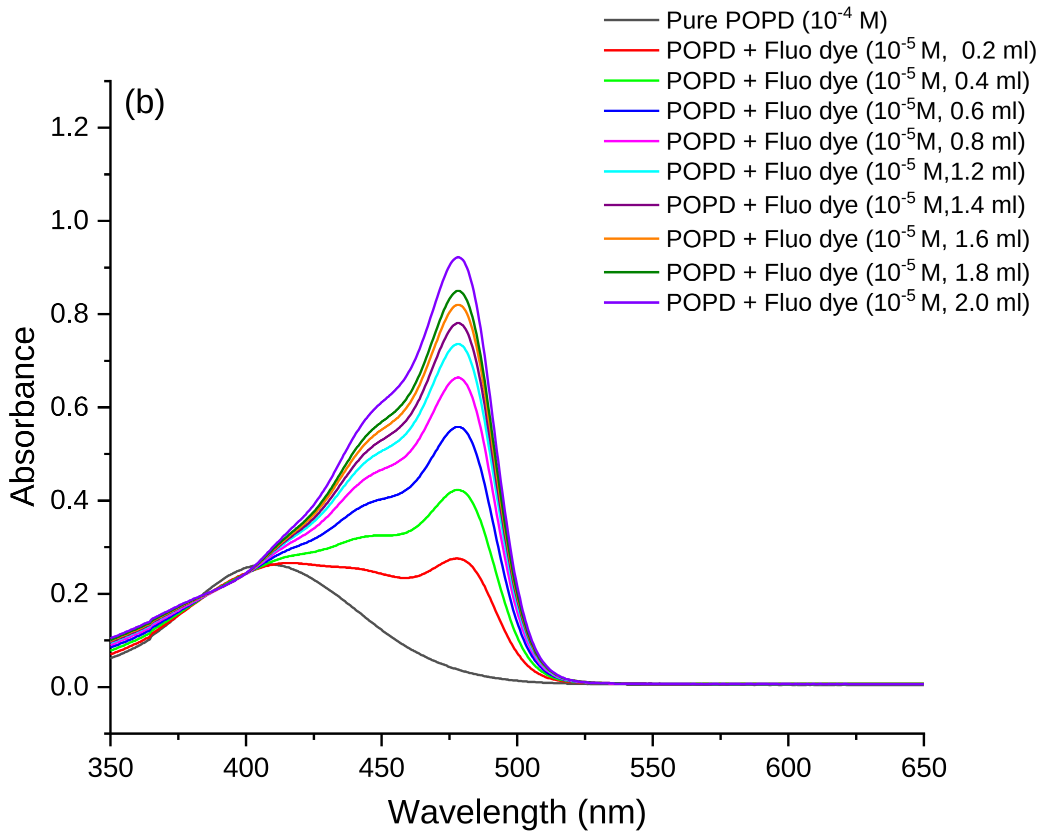
(b)
Pure POPD (10^{-4} M)
POPD + Fluo dye (10^{-5} M, 0.2 ml)
POPD + Fluo dye (10^{-5} M, 0.4 ml)
POPD + Fluo dye (10^{-5}M, 0.6 ml)
POPD + Fluo dye (10^{-5}M, 0.8 ml)
POPD + Fluo dye (10^{-5} M,1.2 ml)
POPD + Fluo dye (10^{-5} M,1.4 ml)
POPD + Fluo dye (10^{-5} M, 1.6 ml)
POPD + Fluo dye (10^{-5} M, 1.8 ml)
POPD + Fluo dye (10^{-5} M, 2.0 ml)
Absorbance
Wavelength (nm)
0.0
0.2
0.4
0.6
0.8
1.0
1.2
350
400
450
500
550
600
650

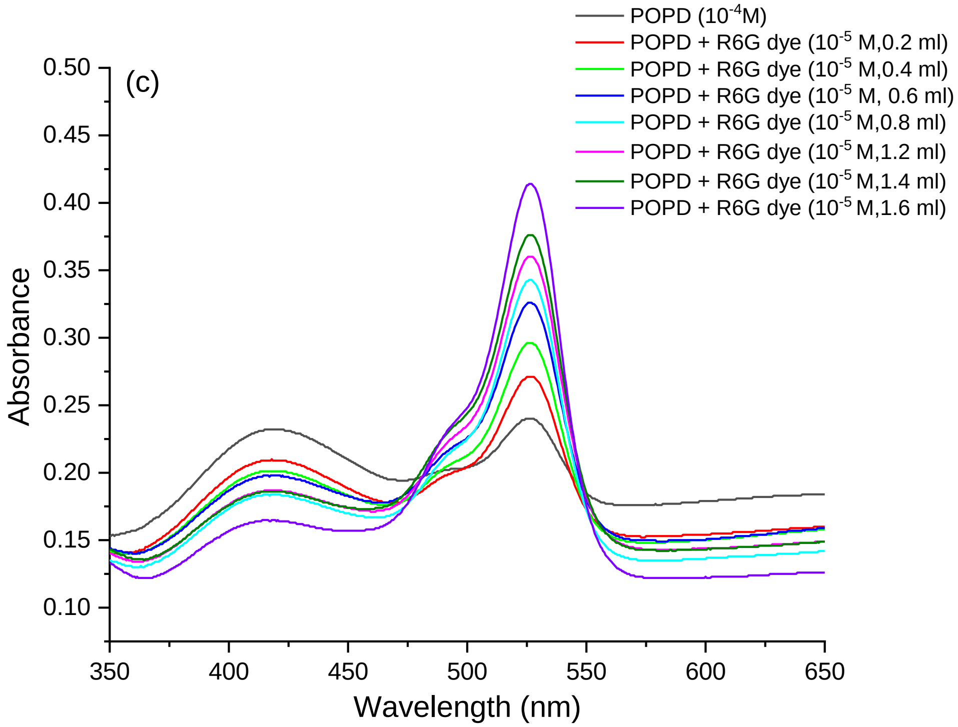

POPD (10-4M)
POPD + R6G dye (10-5 M,0.2 ml)
POPD + R6G dye (10-5 M,0.4 ml)
POPD + R6G dye (10-5 M, 0.6 ml)
POPD + R6G dye (10-5 M,0.8 ml)
POPD + R6G dye (10-5 M,1.2 ml)
POPD + R6G dye (10-5 M,1.4 ml)
POPD + R6G dye (10-5 M,1.6 ml)
(c)
Absorbance
Wavelength (nm)

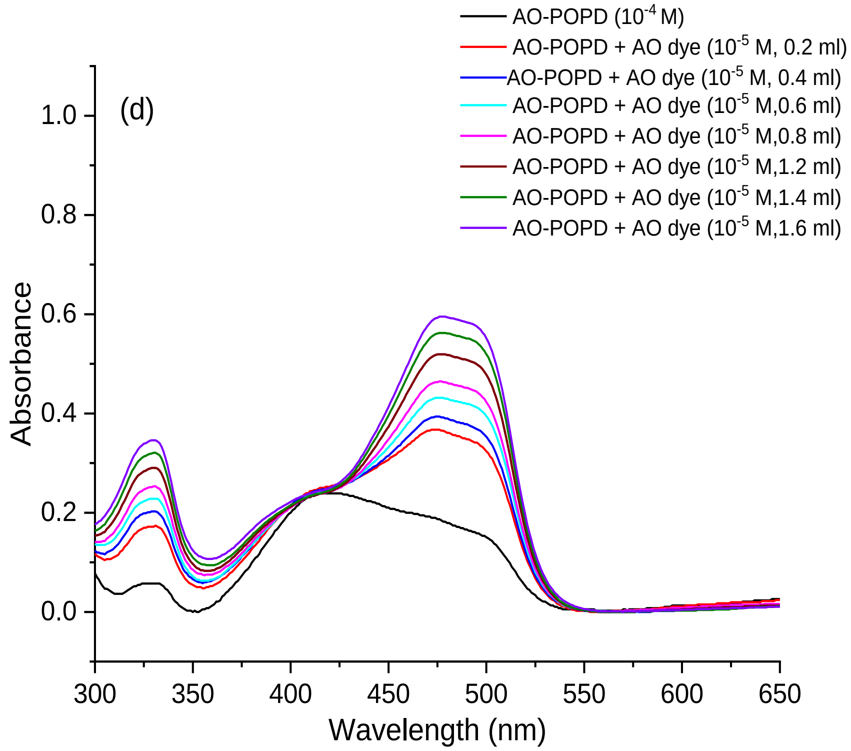

AO-POPD (10-4 M)
AO-POPD + AO dye (10-5 M, 0.2 ml)
AO-POPD + AO dye (10-5 M, 0.4 ml)
AO-POPD + AO dye (10-5 M,0.6 ml)
AO-POPD + AO dye (10-5 M,0.8 ml)
AO-POPD + AO dye (10-5 M,1.2 ml)
AO-POPD + AO dye (10-5 M,1.4 ml)
AO-POPD + AO dye (10-5 M,1.6 ml)
(d)
Absorbance
Wavelength (nm)

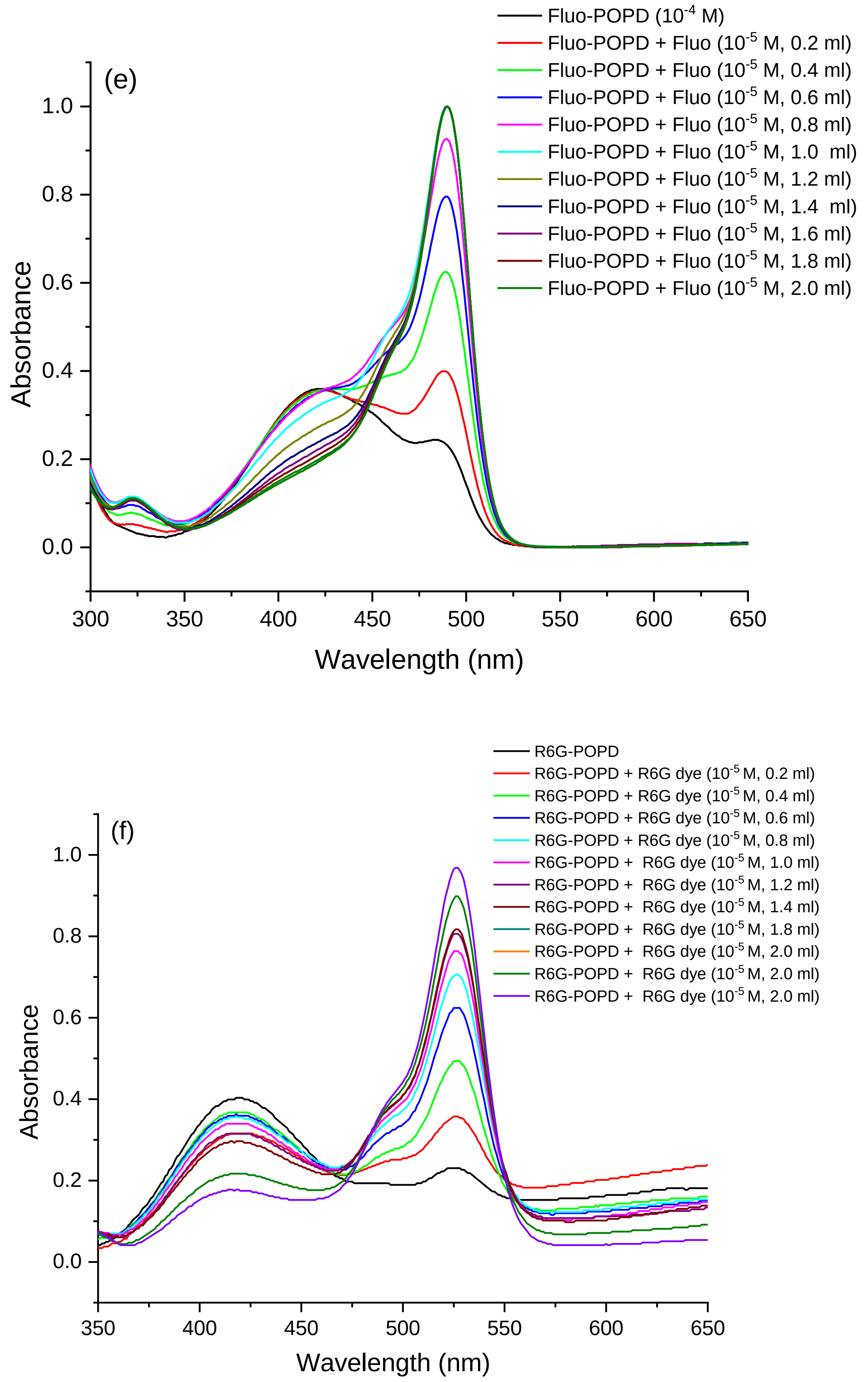

Figure S5 Effect of addition of dye solution to pristine POPD and dye doped POPDs

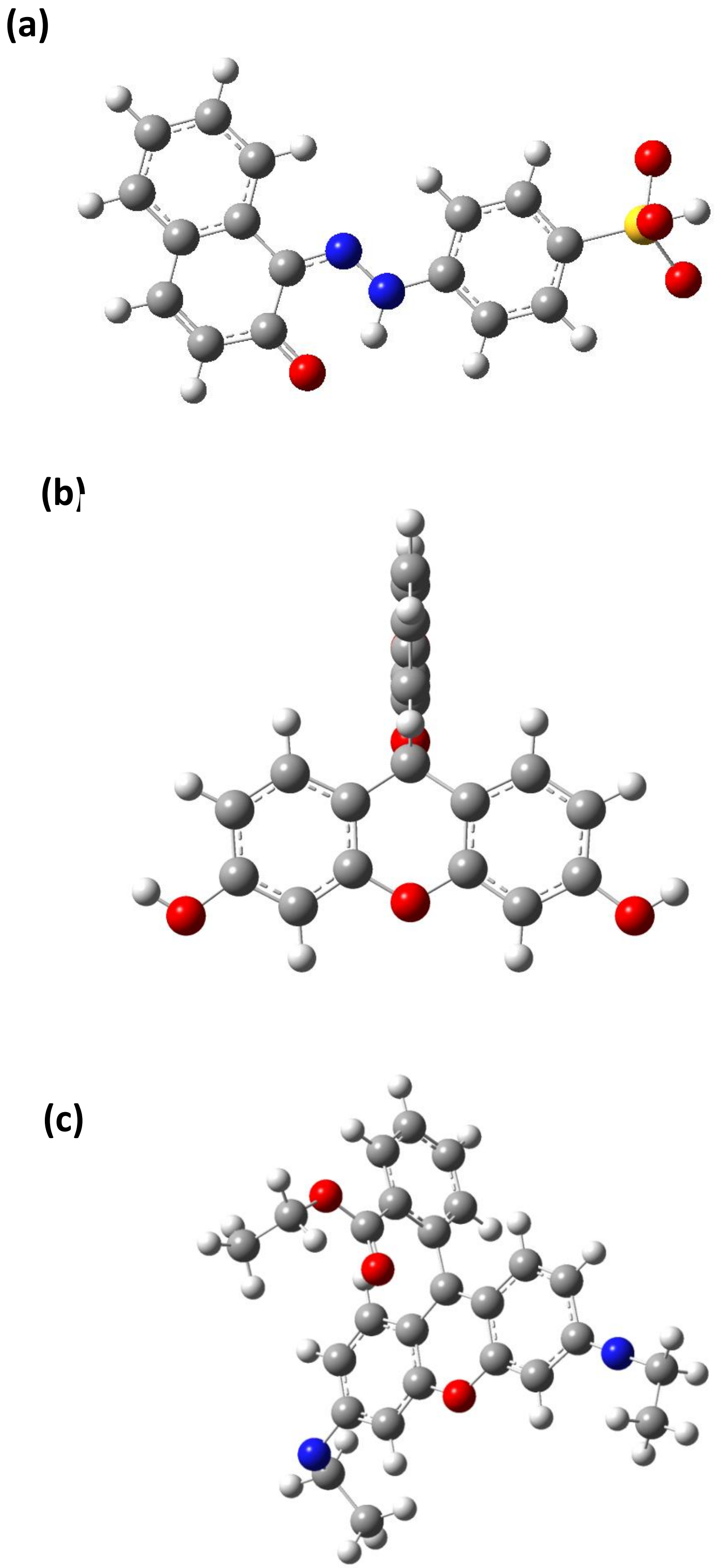

Figure S6 Optimized geometries of (a) AO, (b) Flu and (c) R6G dyes

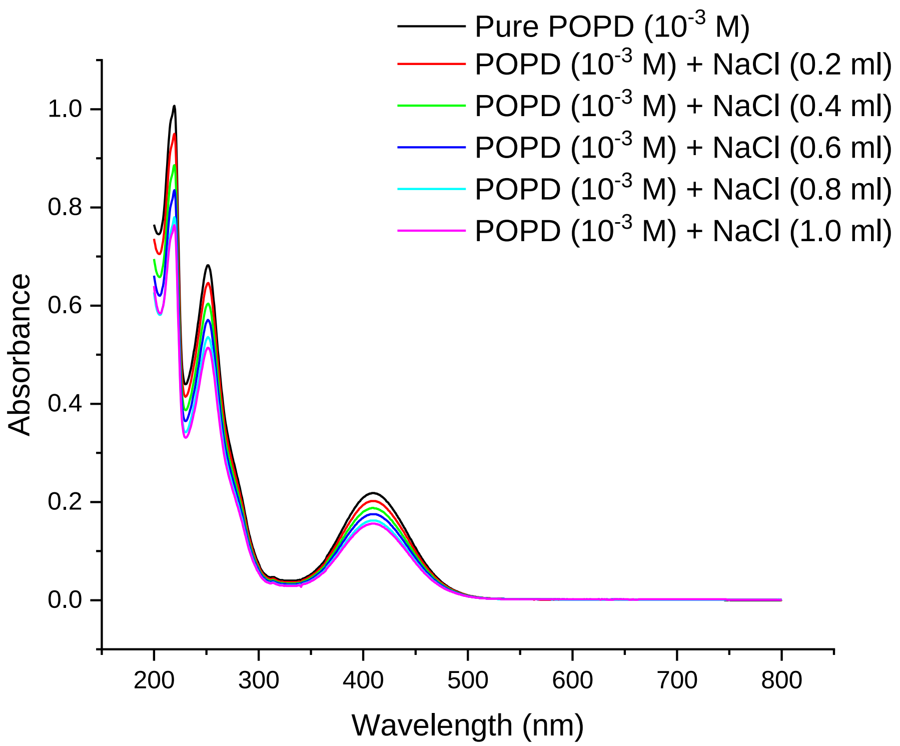

Figure S7 Effect of addition of NaCl to POPD

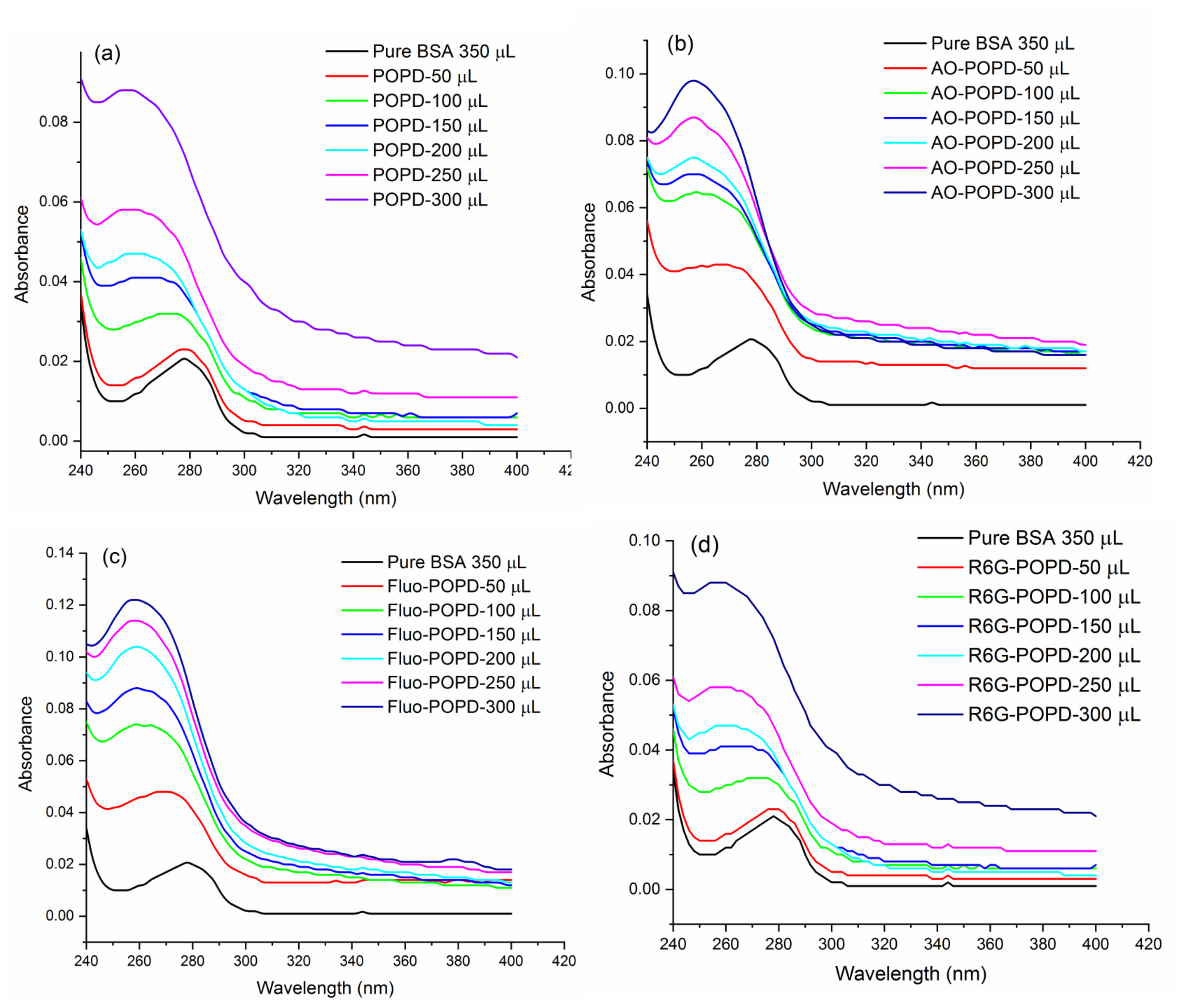

Figure S8 UV spectra of BSA in presence of (a) POPD, (b) AO- POPD,(c) Fluo -POPD, (d) R6G -POPD

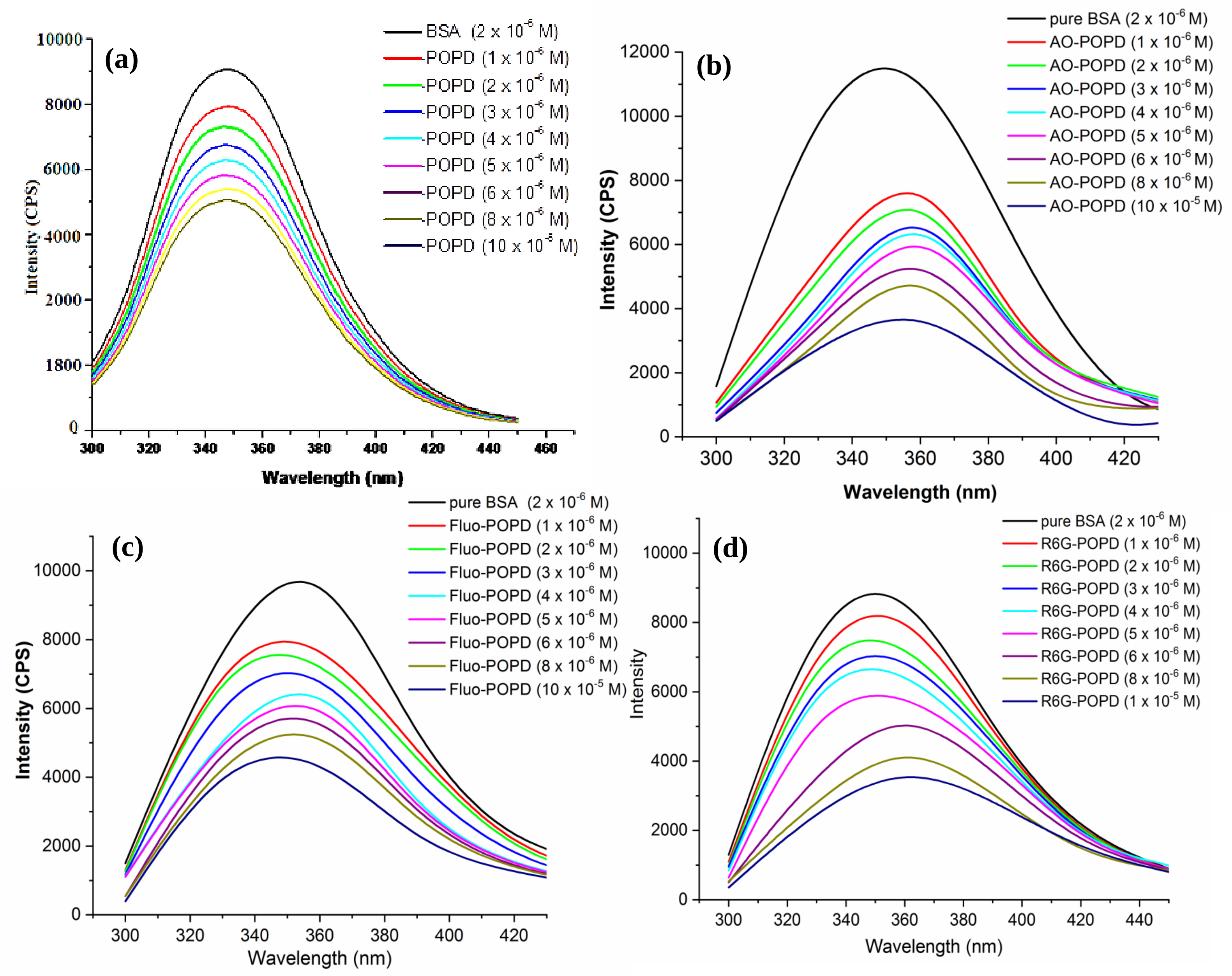

Figure S9 Fluorescence quenching spectra of BSA (λ_{exc}= 280 nm) in presence of (a) POPD, (b) AO-POPD, (c) Fluo-POPD, (d) R6G-POPD

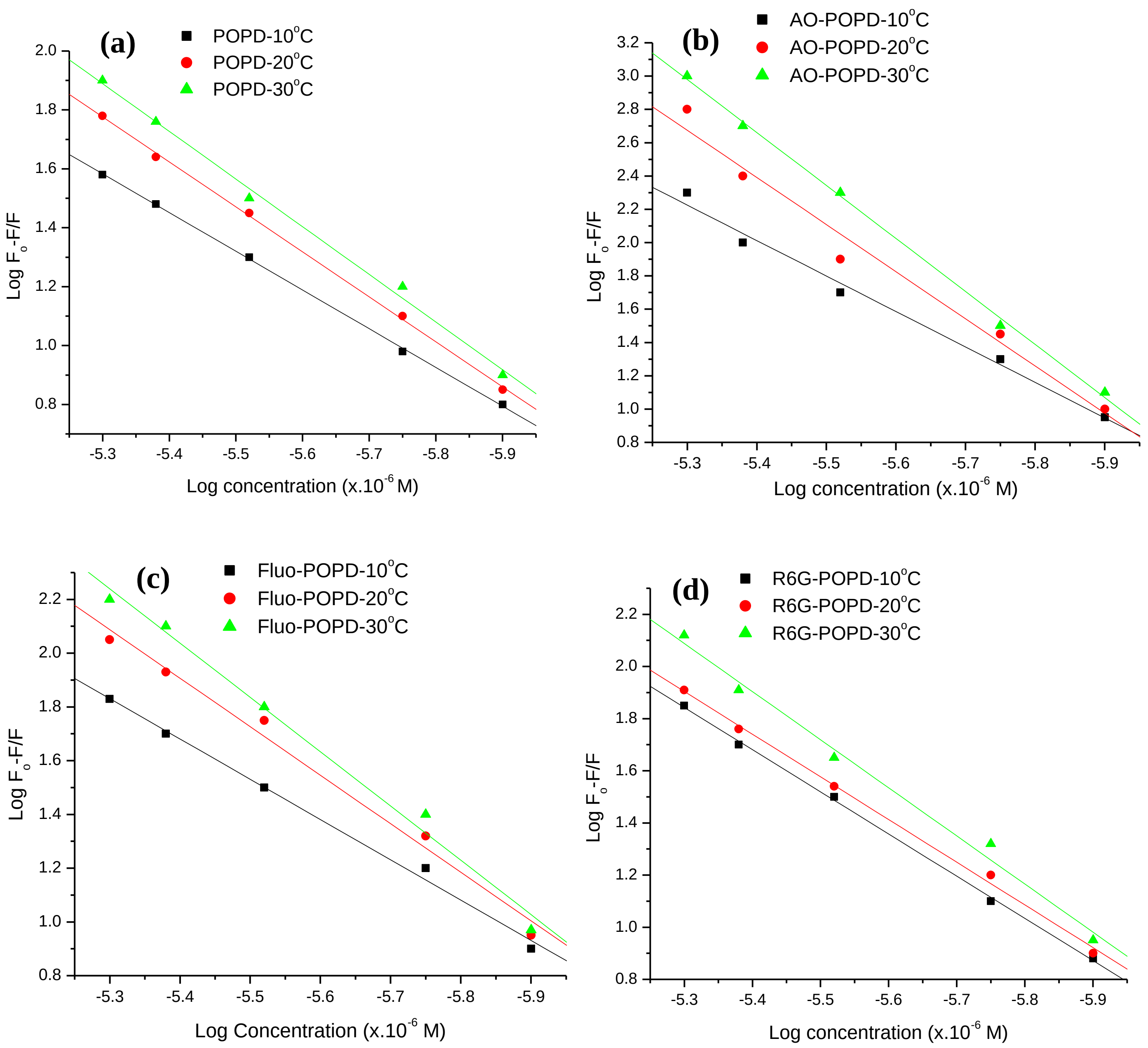

Figure S10 Plot of log F_o-F/F vs log concentration for determination of Ka

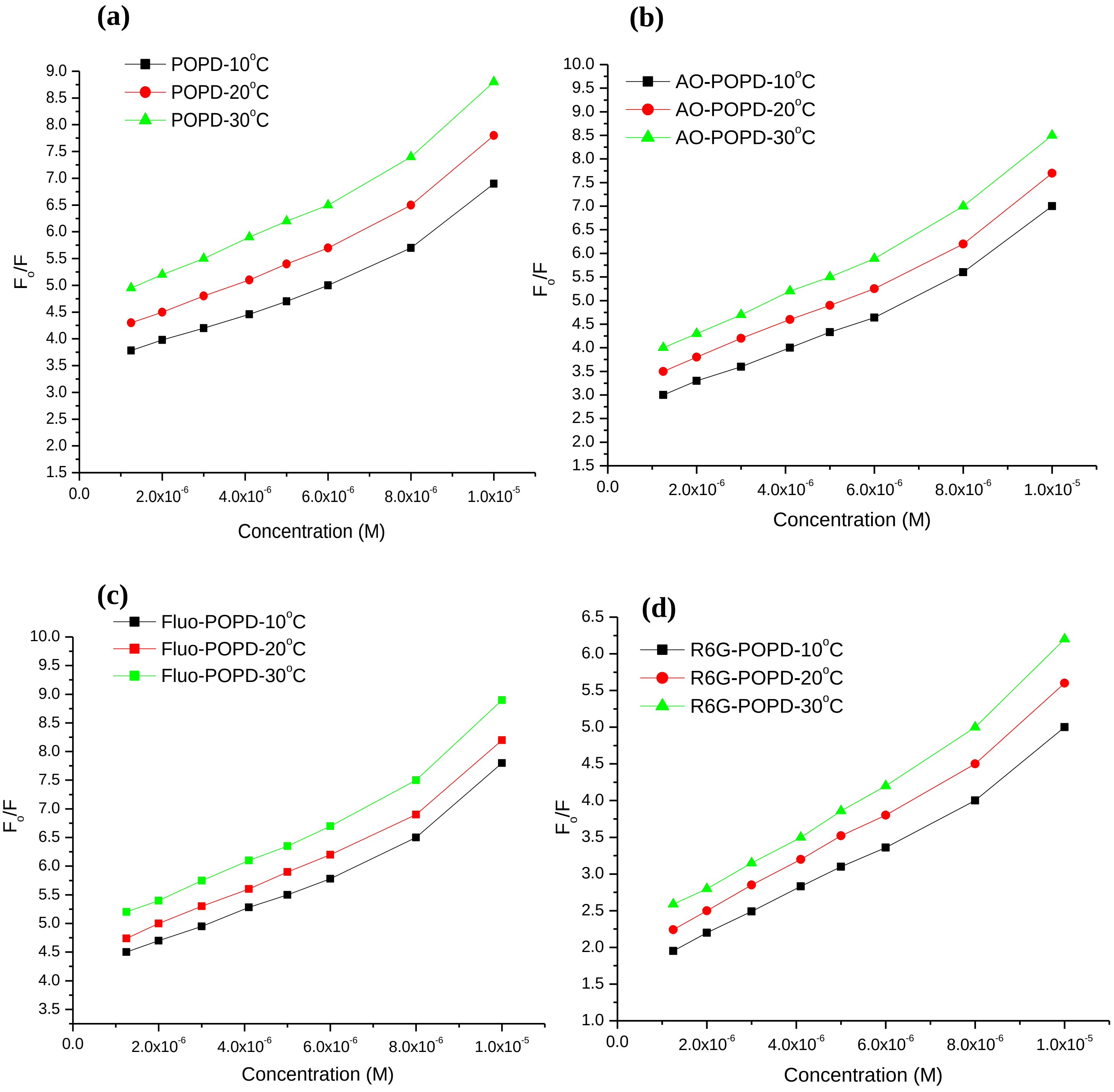

Figure S 11 Stern Volmer plots of (a) POPD, (b) AO- POPD, (c) Fluo- POPD, (d) R6G - POPD

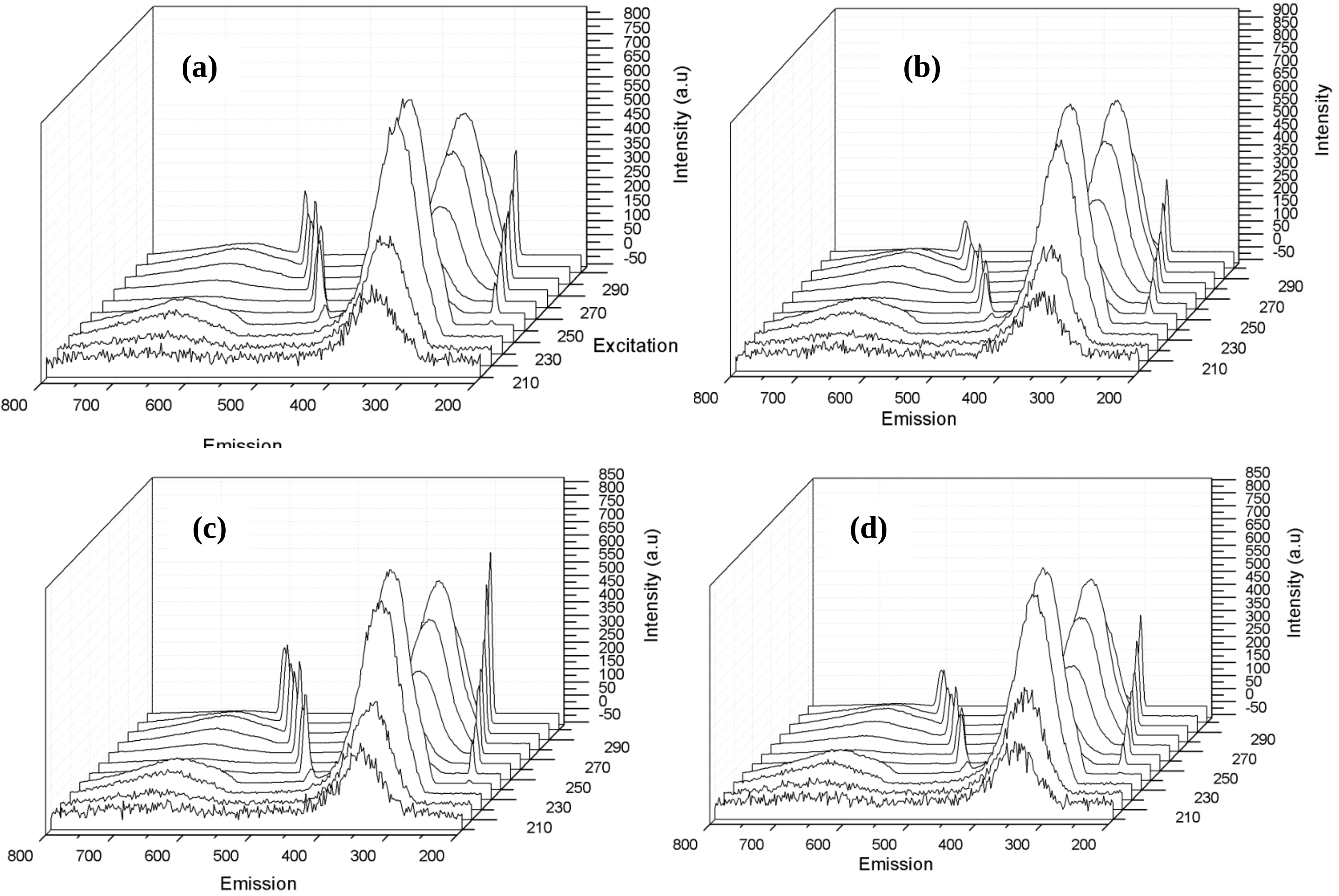

Figure S12 3-D spectra of (a) POPD, (b) AO-POPD, (c) Fluo-POPD (d) R6G-POPD
